# Diblock Copolypeptoid Micelles as Platform for Aqueous Photoredox Cyanation of Arenes

**DOI:** 10.1021/jacs.5c07882

**Published:** 2025-08-04

**Authors:** Afshin Nabiyan, Mitra Esfandiari, Jakob Ruickoldt, Petra Wendler, Nora Kulak, Helmut Schlaad

**Affiliations:** † Institute of Chemistry, 26583 University of Potsdam, Karl-Liebknecht-Str. 24-25, D-14476 Potsdam, Germany; ‡ Institute of Biochemistry and Biology, University of Potsdam, Karl-Liebknecht-Str. 24-25, 14476 Potsdam, Germany

## Abstract

Micellar catalysis offers a sustainable and effective alternative to traditional systems. This study introduces a micellar platform based on the amphiphilic diblock copolypeptoid poly­(*N*-methyl glycine)-*block*-poly­(*N*-n-propyl glycine) for direct arene cyanation in water. The polymer was used to encapsulate the photoredox catalyst 3,6-di-*tert*-butyl-9-mesityl-10-phenylacridinium tetrafluoroborate (Mes-Acr-Ph^+^), forming core–shell nanostructures, as confirmed by dynamic light scattering (DLS) and cryogenic transmission electron microscopy (cryo-TEM). Under visible light irradiation, micellar core confinement activates the catalytic performance of Mes-Acr-Ph^+^, enabling selective C–H cyanation of diverse arenes in water. Recycling experiments demonstrate the stability and reusability of the micelles, highlighting their potential for scalable photoredox applications. This strategy advances aqueous photoredox catalysis by addressing longstanding challenges in catalyst solubility, reactivity, aggregation, close proximity, and reaction control.

## Graphical abstract

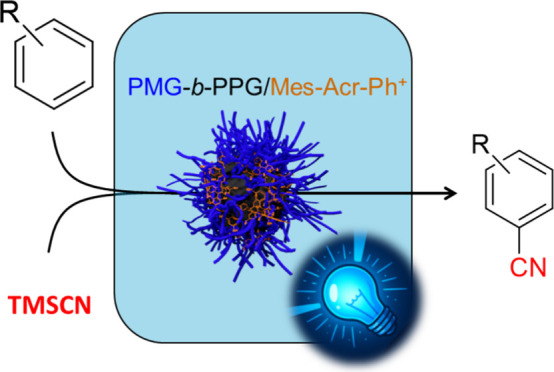

## Introduction

Micelles supporting catalytic processes create well-defined environments, enabling precise control over reaction parameters and enhancing efficiency and selectivity.^1–6^ However, their use in photoredox catalysis remains limited by challenges including light absorption, solubility, structural stability under illumination, and catalyst–substrate interactions.^7–12^ Overcoming these hurdles could advance micellar systems toward sustainable, highly selective photochemical processes for industrial applications.

Over the past decades, photoredox catalysis has advanced significantly in organic synthesis, drawing considerable attention from both academia and industry.^12–16^ It enables the generation of reactive species under mild conditions, facilitating transformations that are otherwise challenging.^13,17,18^ The use of water, an environmentally benign solvent, further enhances the appeal of visible-light photoredox catalysis for organic transformations.^5,13,19–21^ Water not only supports sustainable practices but also offers unique reactivity advantages for photocatalytic centers, such as improving efficiency and versatility.^13,22^ However, fully exploiting water as a solvent in photoredox catalysis remains challenging due to the low solubility of organic compounds and the susceptibility of catalysts and intermediates to decomposition or deactivation in aqueous environments.^20,21^

Inspired by nature, tailored assembly structures have been developed to address the solvent limitations and enhance photoredox efficiency.^9,23^ This has led to the emergence of supramolecular photoredox catalysis, where architectures such as micelles,^4,24^ liposomes,^25,26^ and polymersomes^25^ act as artificial compartments for photoredox reactions.^4,23,25,27–31^ Among the designed structures, micelles, typically formed by low-molecular-weight amphiphiles like sodium dodecyl sulfate (SDS),^32^ polyoxyethanyl-α-tocopheryl succinate (TPGS-750M),^31^ and alkylphenyl-poly­(ethylene glycol) (Triton X-100),^14^ have been most commonly reported for driving photoredox reactions.^4,13^

Micelles offer efficient compartmentalization, recyclability, and stabilization of reactive intermediates under aqueous conditions,^4,13,14,33^ but conventional systems remain sensitive to factors such as concentration, solvent composition, pH, and temperature.^34,35^ Block and multiblock copolymer micelles appear to address some of these limitations.^35–38^ Hereby, they provide enhanced thermodynamic stability, tunable amphiphilicity, and robust self-assembly, enabling the formation of uniform catalytic environments.^29,34–36,39,40^ Through precise control over solubility, dispersibility, and reactant separation,^35,36,39,41–47^ copolymeric micelles can significantly outperform traditional micellar systems.^35,36,39^ Indeed, they have emerged as versatile platforms for supramolecular photoredox catalysis, expanding both the scope and reliability of aqueous photochemical transformations.^29,40,41,48–54^

Integrating block or multiblock copolymers with photoredox-active species often leads to the formation of micelles or colloidal photoredox-active nanoaggregates. Researchers have explored various approaches to construct these nanostructures. Zhang et al.^40^ reviewed strategies for combining photoredox systems with (multi)­block copolymers, mostly through covalent attachment. While covalent attachment provides a stable photoredox framework, it also presents challenges such as limited functionalization efficiency, mass transfer restrictions, and difficulties in reusability and recycling, particularly when photoredox species undergo photobleaching. An excellent alternative but less explored strategy involves the noncovalent encapsulation of small catalytic (or photoredox) molecules within (block) copolymer micelles. Although this approach has received limited attention in photoredox catalysis, it has attracted significant interest in aqueous catalytic reactions, where micelles showed a crucial role in improving reaction conditions and catalyst performance.^55–59^

Alongside synthetic copolymers, natural biomolecules such as (poly)­peptides can form catalytically active micelles that emulate enzymatic efficiency and functionality.^60^ These systems create stable environments that enhance catalytic activity.^29,61–63^ However, peptides often face challenges in forming well-defined micelles due to their propensity to adopt stable secondary structures, such as α-helices and β-sheets, through backbone hydrogen bonding. This structural rigidity limits the flexibility required for dynamic assembly and disassembly processes involved in micelle formation.^64–67^ Polypeptoids—*N*-substituted polyglycines—on the other hand, provide a bioinspired and chemically robust platform suitable for aqueous catalysis. As polypeptoids lack backbone hydrogen bonding, they offer enhanced conformational flexibility and resistance to enzymatic degradation—features that support the formation of stable catalytic micellar systems.^7^ Recent advances have also enabled the synthesis of well-defined linear and cyclic polypeptoids using a variety of robust methodologies.^68–71^ Comprehensive studies have examined their solubility profiles, thermoresponsive behavior, aggregation phenomena, and crystallization in both aqueous media and in the bulk.^71–74^

Being composed of tertiary amide repeating units, polypeptoids eliminate backbone hydrogen bonding and chiral centers, offering distinct advantages for self-assembly and catalytic applications.^7,71,75,76^ Their unique structure also enhance their chemical stability, making them resistant to radicals and nucleophiles, while also providing better control over self-assembly. These biomimetic properties make polypeptoids ideal for advanced catalytic and biological applications. For example, Lipshutz et al.^7^ reported a biodegradable and multifunctional surfactant derived from vitamin E and polysarcosine, which facilitates key organic reactions in medicinal chemistry, such as Suzuki–Miyaura cross couplings, nitro group reductions, and amination.

One functional group that requires attention for reactions in water is the cyano group. Commonly found in natural products and pharmacologically active compounds, the cyano group serves as an important synthetic intermediate. Despite challenges such as poor solubility, selectivity issues, and reagent toxicity, it remains highly versatile, enabling conversion into valuable functional groups like carbamoyls,^77^ carboxyls,^78^ aldehydes,^79^ amines,^79^ and nitrogen-containing heterocycles.^80,81^

Traditional methods for cyanoarene synthesis, such as the Sandmeyer reaction or transition-metal-catalyzed cross-coupling, often require harsh conditions, multiple steps, and lead to poor selectivity.^82–85^ Interestingly, micellar approaches (using low-molecular-weight amphiphiles such as dodecyltetraethylene glycol (Brij 30)) have been explored for green cyanation reactions in aqueous media, typically based on Pd complexes, but they are restricted to reactions involving aryl halides.^86^ However, a promising alternative is photoredox catalysis, as reported by Nicewicz et al.,^80^ where the acridinium catalyst (i.e., 3,6-di-*tert*-butyl-9-mesityl-10-phenylacridinium tetrafluoroborate (Mes-Acr-Ph^+^)) and trimethylsilyl cyanide (TMSCN) are used to synthesize aromatic nitriles under aerobic conditions. Notably, challenges such as strict pH control, reliance on organic solvents, buffers, and reagent toxicity still persist.^80^ These limitations create an opportunity for us to investigate how micellar nanoreactors can transfer photoredox catalysis to water while eliminating key challenges, such as pH control and the need for organic solvents.

Hereby, we introduce a supramolecular system that enables arene cyanation in water. To achieve this, we synthesized a diblock copolypeptoid, poly­(*N*-methyl glycine)-*block*-poly­(*N*-n-propyl glycine) (PMG-*b*-PPG), which undergoes micellization in the presence of Mes-Acr-Ph^+^, encapsulating the photoredox catalyst within the resulting core–shell hybrid micelles. These photoredox-active micelles were evaluated for visible-light-driven cyanation of various arenes, demonstrating high reactivity and selective ortho- and para-cyanation, while eliminating the need for pH control or organic solvents. Our findings suggest that the nanoconfined environment of the micelles in water enhances solubility, stabilizes reactive intermediates, and improves catalytic efficiency, offering a sustainable and effective strategy for aqueous organic synthesis.

## Results and Discussions

### Synthesis and Characterization of Diblock Copolypeptoid and Preparation of Micelles

Amphiphilic diblock copolymers are notable for their ability to self-assemble into well-defined and tunable nanostructures, driven by their covalently linked, compositionally distinct blocks. Typically, stable spherical micelles in aqueous solution are obtained from block copolymers with a hydrophilic mass fraction (*f*_hydrophilic_) greater than 0.45.^87,88^ Accordingly, we synthesized the amphiphilic diblock copolypeptoid PMG_100_-*b*-PPG_38_ (subscripts denote the degree of polymerization (DP) for each block, *f*_hydrophilic_ = 0.66) via a two-step ring-opening polymerization of *N*-alkyl glycine *N*-carboxyanhydrides (NNCAs) (Figure 1A; details in the Supporting Information).^69,71,72^ Hereby, first, the hydrophilic PMG (polysarcosine) block was prepared by polymerizing *N*-methyl glycine-NCA in anhydrous DMF using neopentylamine as the initiator at room temperature for 24 h. After completion, the polymer was precipitated into diethyl ether and dried under vacuum. ^1^H NMR end-group analysis confirmed a DP of 100 for PMG (*M*_n_ 7.2 kg mol^–1^), and size exclusion chromatography (SEC, polystyrene calibration) indicated an apparent number-average molar mass (*M*_n_^app^) of 8.0 kg mol^–1^ and a dispersity (*Đ*) of 1.2 (Figure S1). In the second step, the PMG_100_ was used as a macroinitiator to polymerize the *N*-n-propyl glycine-NCA under similar conditions for 48 h, yielding the hydrophobic PPG block. ^1^H NMR analysis of the isolated diblock copolypeptoid revealed a molar fraction of n-propyl glycine of 0.27, corresponding to a DP of 38 for the PPG block, and thus *M*_n_ 10.9 kg mol^–1^; SEC: *M*_n_^app^ 11.0 kg mol^–1^, *Đ* 1.1 (Figure S2).

To explore the self-assembly behavior, the PMG_100_-*b*-PPG_38_ was first dissolved in methanol, a nonselective solvent for both blocks, followed by the gradual addition of water, a selective solvent for PMG, to induce micellization. Dialysis against water removed the methanol, yielding stable copolypeptoid micelles (Figure 1B) with an average hydrodynamic diameter of ∼ 38 nm (DLS) and spherical shape (TEM and cryo-TEM) (Figures 1C–D and Figures S3–S4).

**Figure 1:**
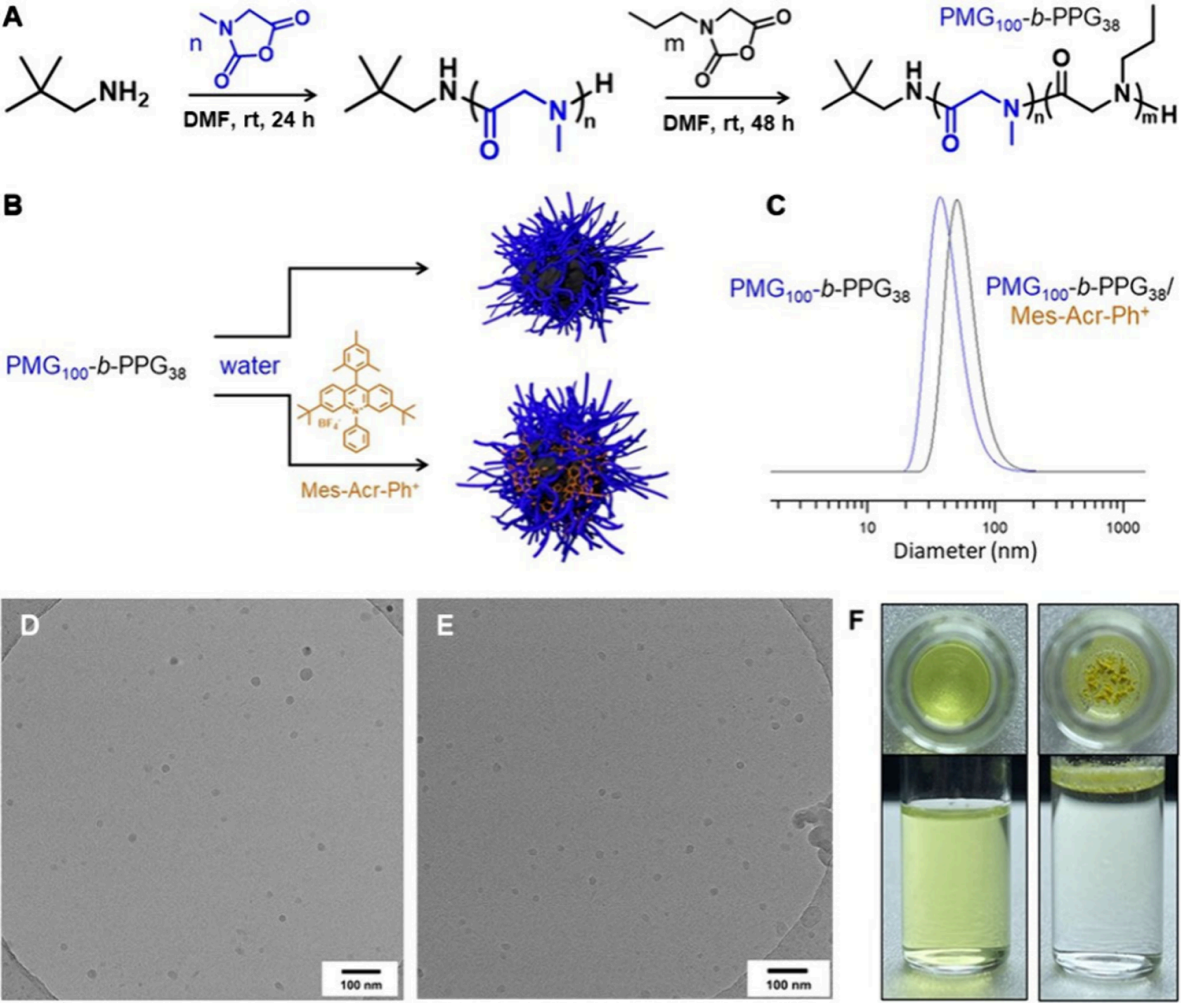
(A) Schematic representation of the synthesis of PMG_100_-*b*-PPG_38_ diblock polypeptoid by ring-opening polymerization of *N*-alkyl glycine *N*-caboxyanhydrides (NNCAs). (B) Schematic illustration of the formation of PMG_100_-*b*-PPG_38_ and PMG_100_-*b*-PPG_38_/Mes-Acr-Ph^+^ micelles in water. (C) DLS size distribution by intensity of PMG_100_-*b*-PPG_38_ and PMG_100_-*b*-PPG_38_/Mes-Acr-Ph^+^ micelles in water. (D, E) Cryo-TEM micrographs of (D) PMG_100_-*b*-PPG_38_ micelles (3 mg/mL) and (E) PMG_100_-*b*-PPG_38_/Mes-Acr-Ph^+^ micelles (4 mg/mL). (F) Optical photographs of a homogeneous aqueous dispersion of PMG_100_-*b*-PPG_38_/Mes-Acr-Ph^+^ micelles (left) and free Mes-Acr-Ph^+^ in water (right) (top and side view).

Acridinium derivatives have broad solubility in organic solvents, but their solubility in water is limited (as macroscopic particles are observed in water, see Figure 1F (right)).^89^ Thereby, to address this, we prepared Mes-Acr-Ph^+^-loaded micelles by dissolving Mes-Acr-Ph^+^ and PMG_100_-*b*-PPG_38_ in methanol, followed by gradual addition of water to induce micellization. The resulting homogeneous PMG_100_-*b*-PPG_38_/Mes-Acr-Ph^+^ dispersion exhibited a distinct yellowish tint (Figure 1F (left)), unlike the free Mes-Acr-Ph^+^ in water, indicating successful encapsulation within the micellar PPG core. The ultraviolet–visible (UV–vis) spectrum of Mes-Acr-Ph^+^ encapsulated inside the micellar core closely resembled that of Mes-Acr-Ph^+^ in acetonitrile although with a slight redshift (Figure S5). This redshift is probably due to a solvatochromic effect, where the dye experiences a less polar environment within the hydrophobic micellar core as compared to the polar acetonitrile solvent. Such environmental changes likely influence the electronic transitions of the dye, leading to a bathochromic shift in the absorption spectrum. DLS (Figure 1C), TEM and cryo-TEM analyses (Figure 1E and Figures S6–S7) confirmed the formation of well-defined spherical nanoaggregates of PMG_100_-*b*-PPG_38_/Mes-Acr-Ph^+^ measuring about 43 nm in diameter (DLS) (and thus are slightly larger than the original PMG_100_-*b*-PPG_38_ micelles, see above).

### Light-Driven Cyanation of Arenes by Photoredox Active-Micelles

Inspired by Nicewicz’s work,^84^ we first investigated the cyanation of 2,6-dimethoxypyridine (2,6-DMP) as a model substrate with trimethylsilyl cyanide (TMSCN) in Milli-Q water (pH ∼ 6) under an oxygen atmosphere at room temperature. Reactions were performed in a custom-made photoreactor with a LED visible light source (Supporting Information, λ_max_ = 450 nm), using the acridinium catalyst both alone and confined within PMG_100_-*b*-PPG_38_ micelles (Table 1).

**Table 1:**
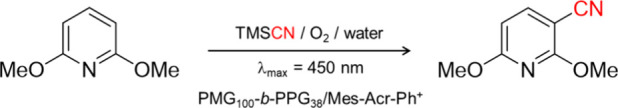
Optimization of Reaction Conditions for the Cyanation of 2,6-DMP With TMSCN Using PMG_100_-*b*-PPG_38_/Mes-Acr-Ph^+^ Micelles in Water

Entry^a^	Atm.^b^	pH	TMSCN/2,6-DMP^e^	Conversion (%)^f^
1	O_2_	∼6^c^	1.3	69
2	Ar	∼6^c^	1.3	0
3	Air	∼6^c^	1.3	10
4	O_2_	8–9^d^	1.3	80
5	O_2_	8–9^b^	2.6	>95
6	O_2_	8–9^d^	3.9	90
7	O_2_	8–9^d^	5.2	90

a Reactions performed using PMG_100_-*b*-PPG_38_/Mes-Acr-Ph^+^ at a total concentration of 0.2 mg/mL, irradiated with a 450 nm LED lamp for 24 h.

b Atmospheres (O_2_, Air, or Ar) were purged for 15 min prior to reaction.

c Milli-Q water was used without pH adjustment.

d pH was adjusted using saturated NaOH solution.

e Molar ratio of TMSCN to 2,6-DMP (0.03 mM).

f Conversion of 2,6-DMP determined by ^1^H NMR analysis of the crude reaction mixture, comparing the peak integrals of methoxy groups to HMDSO as internal standard.

As anticipated, in the absence of micellar encapsulation, Mes-Acr-Ph^+^ exhibited poor solubility and pronounced aggregation in water, severely limiting light absorption and rendering the photocatalyst inactive for cyanation under visible-light irradiation (see above, solution behavior). Instead, the PMG_100_-*b*-PPG_38_/Mes-Acr-Ph^+^ micellar system afforded a 69% conversion of 2,6-DMP after 24 h (Table 1, entry 1), as determined by ^1^H NMR analysis of the crude mixture using the depletion of the methoxy signal at δ 3.84 ppm relative to hexamethyldisiloxane (HMDSO) as an internal standard. This result indicates that micellar confinement effectively prevents catalyst aggregation, ensuring uniform dispersion of Mes-Acr-Ph^+^ and enabling productive photoredox activity. The 2,6-dimethoxynictotinonitrile product was afterward isolated by column chromatography and identified by ^1^H and ^13^C NMR spectroscopy and GC-MS (see Supporting Information, Figures S8–S9). Further control experiments showed that the cyanation reaction did neither occur in the dark nor in the absence of oxygen (Table 1, entry 2). Notably, replacing oxygen with ambient air drastically reduced the conversion to 10% (Table 1, entry 3).

Previous studies by Nicewicz,^80,84^ also demonstrated that cyanation efficiency is highly sensitive to the reaction medium. Mixed organic–aqueous systems, such as acetonitrile with NaHCO_3_ or phosphate buffers at basic pH (∼9) showed significantly improved results. Motivated by these observations, we explored the effect of pH and buffering conditions in our fully aqueous micellar system. Interestingly, the PMG_100_-*b*-PPG_38_/Mes-Acr-Ph^+^ micelles exhibited reduced activity in phosphate buffer (Table S1) but improved activity at pH > 8 (Table 1, entry 4; Table S1). However, unlike the Nicewicz system, further increasing the pH beyond pH 10 did not significantly reduce the reaction efficiency. This observation may be related to the pH-sensitive hydrolysis of TMSCN in strongly acidic and basic media and its conversion to cyanate species, where both pH and buffer conditions govern cyanide release.^80,90,91^ In our system, the TMSCN and Mes–Acr–Ph^+^ are encapsulated within the micellar core, shielding them from the aqueous phase. This hydrophobic confinement enables a more gradual and sustained release of CN^–^ and may also localize it near the arene radical cation, promoting efficient coupling. Moreover, the micellar environment may prevent oxidation of cyanide to cyanate.^80,92^ As a result, our system achieves efficient C–H cyanation even under unbuffered conditions or high pH. These features suggest that the micellar nanoreactor not only enhances reactivity but also reduces the need for strict pH control or buffering agents, offering a robust and practical platform for aqueous photoredox cyanation.

Further optimization experiments (Table 1, entries 4–7) revealed that the highest conversion of >95% was achieved at a TMSCN/2,6-DMP molar ratio of 2.6. Notably, this conversion was reached within just 15 h (Figure 2). Slightly lower conversions were observed when TMSCN was used in larger excess. Interestingly, increasing the concentration of 2,6-DMP beyond 0.1 mM led to the formation of two phases and change in color from yellow to colorless—likely due to catalyst decomposition or precipitation. Although the critical micelle concentration (CMC) should be very low, on the order of 10^–6^ M,^93^ ensuring micelle stability even under dilute conditions, the micelles appeared to be disrupted at higher 2,6-DMP concentrations. This is likely due to the dynamic nature of the micelles and the enhanced solubility of the Mes-Acr-Ph dye in the organic-rich environment containing excess 2,6-DMP. This may facilitate leakage of the catalyst from the micellar core, leading to biphasic behavior as the micelles break apart. Collectively, these findings demonstrate that the micellar platform not only enhances catalyst solubility but also provides a tunable aqueous microenvironment that supports efficient C–H cyanation without the need for organic solvents, external pH control, or phosphate buffers, representing a significant step toward fully water-based photoredox catalysis.

**Figure 2:**
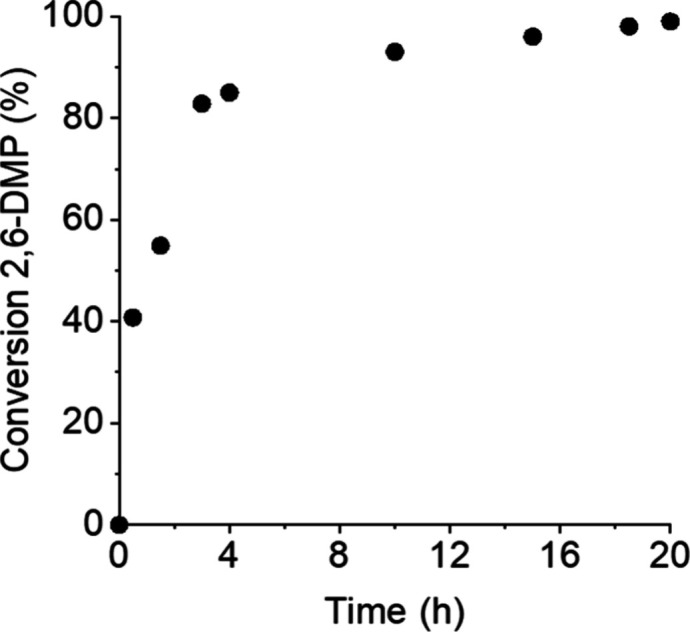
Time–conversion plot for the reaction of 2,6-DMP with TMSCN using PMG_100_-*b*-PPG_38_/Mes-Acr-Ph^+^ micelles in aqueous solution, referring to entry 5 in Table 1.

### Exploring the Scope of Photoredox Cynation

Various arenes, including mono-, di-, and trisubstituted arenes as well as biphenyl derivatives, were examined to assess the versatility of the CN transformation with the PMG_100‑_ *b*-PPG_38_/Mes-Acr-Ph^+^ photoredox catalyst (Table 2 and Table S2).

**Table 2:**
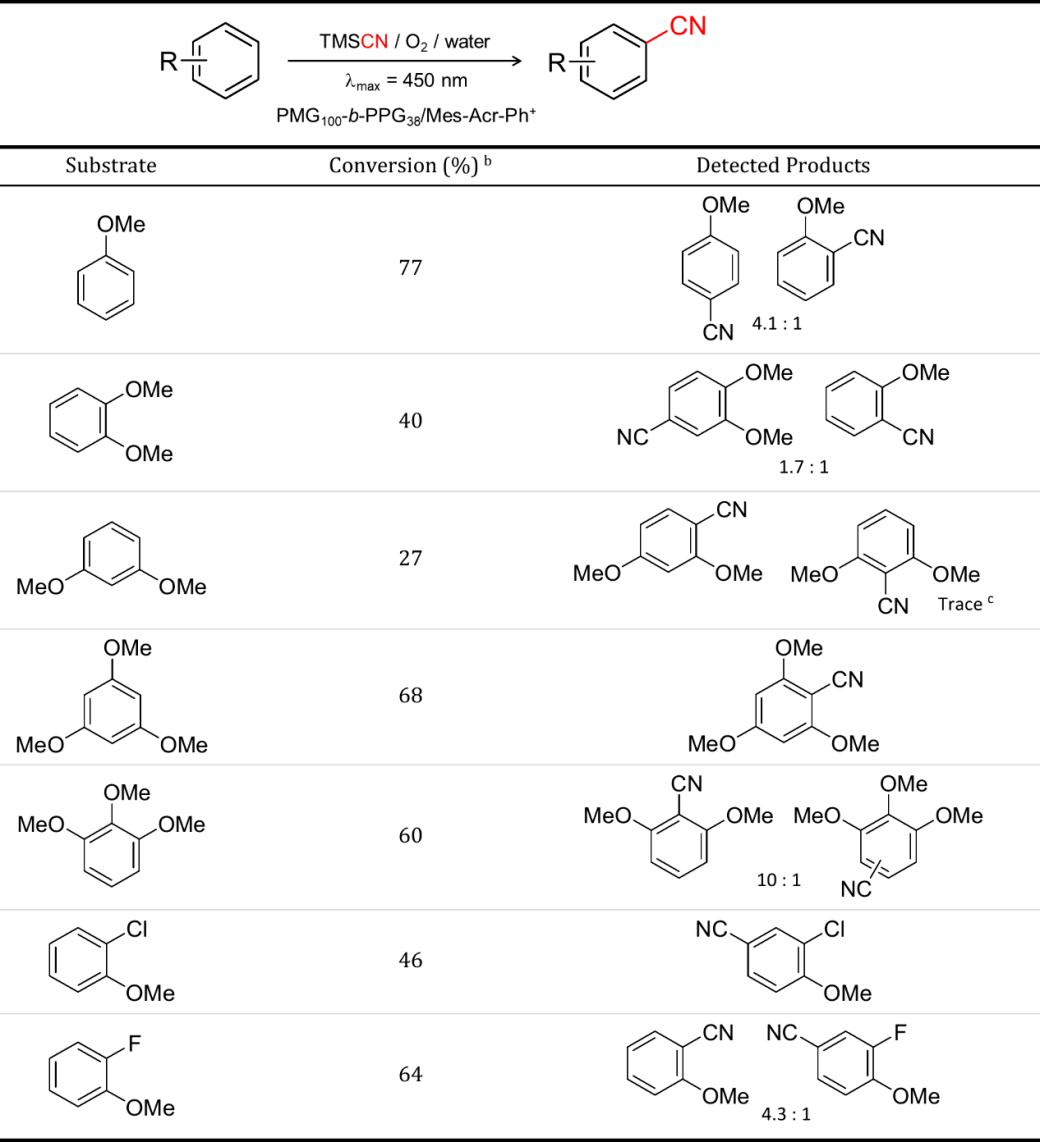
Scope of Arene Cyanation Reaction by PMG_100_-*b*-PPG_38_/Mes-Acr-Ph^+^ Micelles in Water

a Reactions were conducted using 5 mL of a 0.2 mg/mL solution of PMG_100_-*b*-PPG_38_/Mes-Acr-Ph^+^, arene substrate with concentration of 0.06 mM and molar ratio TMSCN/arene = 1.3. Irradiation with LED lamp (λ_max_ = 450 nm) for 20 h. O_2_ purged for 15 min.

b Conversion was obtained from the crude reaction mixture by ^1^H NMR spectroscopy (comparing the peak integrals of methoxy groups to HMDSO as internal standard).

c Trace amounts of product were detected exclusively by GC-MS.

Among the mono-, di-, and trisubstituted arenes, those bearing methoxy groups (Table 2, entries 1–5) consistently afforded the highest conversions to the corresponding benzonitrile products. For instance, anisole, as a monosubstituted arene (entry 1), underwent conversion to mixtures of regioisomers, predominantly *meta*- and *para*-substituted benzonitriles, indicating limited regiocontrol in the absence of strong directing groups. Similarly, arenes substituted with 2-chloro and 2-fluoro groups (Table 2, entries 6 and 7) displayed moderate to good conversions, further demonstrating the system’s tolerance toward electron-withdrawing substituents.

Our catalytic activity studies reveal that substrates such as mesitylene, biphenyl, and 2-methoxynaphthalene (Table S2) exhibited significantly lower conversions, likely due to poor solubility and limited partitioning into the micellar phase. Specifically, biphenyl and methoxybiphenyl, which are solid at room temperature, underwent only trace conversion, reflecting their limited compatibility with the aqueous micellar environment. Additionally, mesitylene, a trisubstituted arene lacking methoxy groups, predominantly gave overoxidation products, including aldehydes, highlighting the reduced reactivity of these substrates under the given conditions.

Substrates bearing 1,3,5- and 1,2,3-trimethoxy substitution patterns (Table 2, entries 4 and 5) underwent efficient transformation to the corresponding trimethoxy benzonitriles. In the case of the 1,2,3-trimethoxy, a demethylated product retaining only two methoxy groups was also detected, indicating simultaneous C–H and *ipso*-C–O cyanation under identical conditions. A similar dual reactivity emerged with the 1,2-dimethoxy substrate (Table 1, entry 2), highlighting the propensity of ortho-methoxy groups to engage in *ipso* substitution. Together, these results showcase the scope and selectivity of micellar catalysis for aqueous arene cyanation and highlight the influence of electronic structure, substitution pattern, and solubility on product outcomes.

### Recycling Ability and Photoredox Mechanism of PMG_100_-*b*-PPG_38_/Mes-Acr-Ph^+^ Micelles

The PMG_100_-*b*-PPG_38_/Mes–Acr–Ph^+^ micelle system was tested for the cyanation of 2,6-DMP over multiple cycles. A gradual decrease in catalytic performance was observed from the second to the fourth cycle, accompanied by a visible color change in the dispersion (Figure 3A).

**Figure 3:**
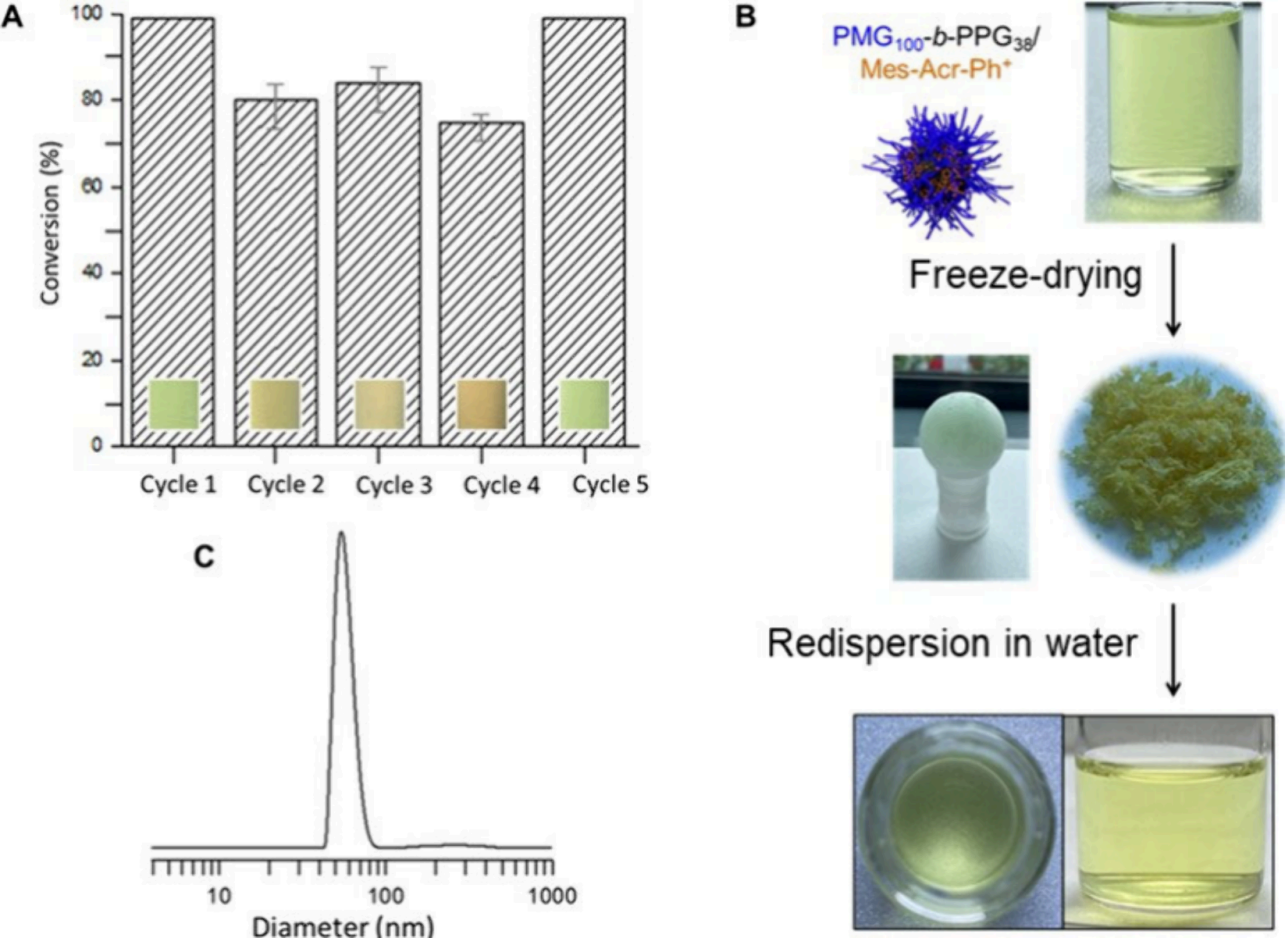
(A) Conversion of 2,6-DMP to 2,6-dimethoxynicotinonitrile over multiple cycles using PMG_100_-*b*-PPG_38_/Mes-Acr-Ph^+^ micelles, TMSCN (0.03 mM), and 2,6-dimethoxypyridine (0.02 mM) under visible light, with cycle 5 involving fresh Mes-Acr-Ph^+^ loaded micelles after dialysis. Bottom: Optical images of the reaction mixtures under the specified conditions. (B) Illustration of the preparation and dispersion of PMG_100_-*b*-PPG_38_/Mes-Acr-Ph^+^ before and after freeze-drying. (C) DLS size distribution by intensity of PMG_100_-*b*-PPG_38_/Mes-Acr-Ph^+^ micelles in aqueous solution, obtained after redispersing the freeze-dried sample.

The observed decline in catalytic performance over successive cycles is likely due to partial release and/or degradation of the Mes-Acr-Ph^+^ photoredox catalyst from the micellar core. Indeed, once released into the aqueous phase, Mes-Acr-Ph^+^ may undergo aggregation into catalytically inactive forms. However, photodegradation appears to be the predominant cause of deactivation. Hereby, GC–MS analysis of the postreaction mixture revealed signals consistent with acridinium degradation derivatives including oxidized and fragmented species (Figure S21). This finding is further supported by the progressive color fading of the solution (e.g., from yellow to colorless) observed under extended irradiation. Although catalyst leakage from the micelle cannot be fully excluded, its contribution is likely negligible and more difficult to disentangle from photodegradation. These results are consistent with prior reports highlighting the limited photostability of acridinium dyes under photoredox conditions.^94–98^

To address the deactivation of Mes-Acr-Ph^+^ and restore the system’s efficiency, we disrupted the micelles by dissolving them in methanol (or acetonitrile) and removing the residual or degraded Mes-Acr-Ph^+^ by dialysis against a water/methanol (or water/acetonitrile) mixture. The micelles were then reformed and reloaded with a fresh batch of Mes-Acr-Ph^+^. The refreshed micellar system exhibited a catalytic performance comparable to the initial cycle (>90% conversion), highlighting its robustness and recyclability (Figure 3A cycle 5).

In parallel, we explored a simpler approach by freeze-drying the aqueous micelle dispersion. The resulting yellow powder could be readily redispersed in water to regenerate a homogeneous dispersion (Figure 3B) containing ∼50 nm particles, similar in size to the original system (DLS, Figure 3C). The redispersed micelles retained over 90% of their catalytic activity, as confirmed by a benchmark reaction under standard conditions (Table 1, entry 5).

DLS analysis of samples recovered by both dialysis and freeze-drying showed negligible differences in hydrodynamic diameter and size distribution, indicating that the micellar structure and integrity were preserved during these recovery steps. This ability to store, reuse, and maintain performance without significant loss enhances the practicality and user-friendliness of the micellar catalytic system for broader applications.

The electrochemical potential of a photocatalyst is crucial in photoredox reactions, as it determines the catalyst’s ability to facilitate specific electron transfer processes with substrates and intermediates, thereby influencing the efficiency and selectivity of the reaction. However, the photoredox potential is solvent-dependent, with notable discrepancies when water replaces organic solvents as the reaction medium.^99^ To examine the influence of solvent and micellar confinement on the redox potential of Mes-Acr-Ph^+^, we conducted cyclic voltammetry (CV) on PMG_100_-*b*-PPG_38_/Mes-Acr-Ph^+^ micelles in water using low-cost, disposable screen-printed carbon electrodes (SPCEs) to facilitate reliable electrochemical characterization.

The resulting cyclic voltammogram displayed a reversible characteristic with a distinct duck-shaped profile. Both the anodic and cathodic peak currents exhibited a linear dependence on the square root of the scan rate, suggesting a diffusion-controlled electrochemical process (Figure 4A and Figure S20).^100^ The ground-state redox potential, measured from a 3 mg/mL micellar solution, was *E*_1/2_ = −0.55 V vs Ag/AgCl (Figure 4A), closely matching the reported value in organic solvents (−0.56 V vs Ag/AgCl).^96^ It therefore appears that the potential characteristics of Mes-Acr-Ph^+^ remain largely unaffected in micelles and water, that is why we propose a mechanism for the cyanation reaction with PMG_100_-*b*-PPG_38_/Mes-Acr-Ph^+^ micelles as reported by Nicewicz et al. (Figure 4B).^84,101^ Briefly, under blue light irradiation, the photocatalyst is excited to its highly oxidizing state ((Mes-Acr-Ph^+^)*, E_1_/_2_ red* = +2.15 V vs SCE) and oxidizes the arene to generate a radical cation, reducing itself to Mes-Acr-Ph^•^. The cyanide anion selectively adds to the arene radical cation at the ortho or para position, forming a cyclohexadienyl radical intermediate. This intermediate is oxidized by molecular oxygen or hydroperoxy radicals to yield the benzonitrile product, with these oxidants regenerating Mes-Acr-Ph^+^ to close the catalytic cycle. However, substrates bearing phenolic, amino, or acidic functional groups, such as phenols or benzoic acids, were found to be incompatible with this catalytic mechanism, resulting in no product formation. This is consistent with the known oxidative instability of such motifs in acridinium photoredox catalysis and likely arises from mismatched redox potentials or competing side reactions such as catalyst quenching and overoxidation. These limitations reflect the inherent constraints of the Mes-Acr-Ph^+^ photocatalyst rather than the micellar system. Expanding the substrate scope beyond electron-rich arenes remains a key objective, and we believe that micellar media may eventually offer solutions through localized encapsulation or buffering effects.

**Figure 4:**
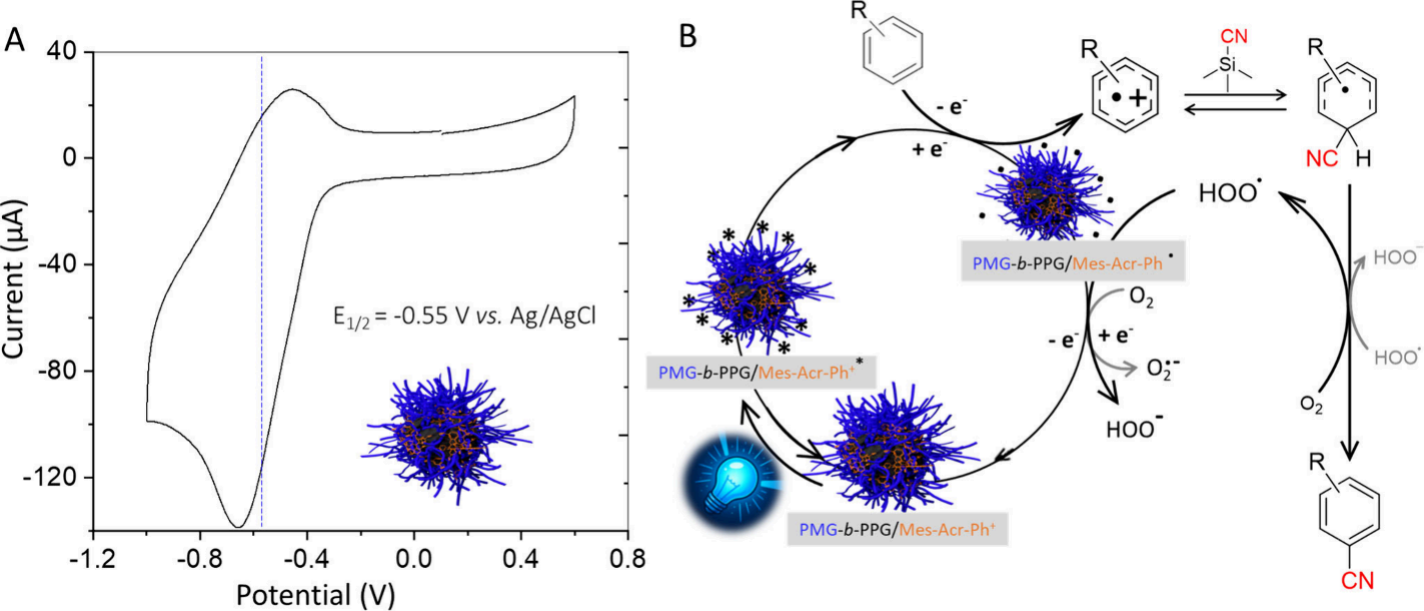
(A) Cyclic voltammogram of an aqueous solution of PMG_100_-*b*-PPG_38_/Mes-Acr-Ph^+^ micellar photoredox catalyst (3 mg/mL)/ KCl (100 mM) on SPCE; scan rate: 100 mV·s^–1^; potential range: −0.1 to +0.6 V (vs Ag/AgCl). (B) Proposed mechanism for photoredox-mediated generation of benzonitriles by PMG_100_-*b*-PPG_38_/Mes-Acr-Ph^+^ micellar photocatalyst.

## Conclusion

In summary, we report a photoredox-active micellar system based on the diblock copolypeptoid PMG_100_-*b*-PPG_38_ that enables visible-light-driven arene cyanation in water using Mes-Acr-Ph^+^. Our observations on morphology and solution behavior confirm that the micelles self-assemble into core–shell nanostructures and encapsulate the photocatalyst within their hydrophobic cores, generating confined catalytic environments. These photoredox-active micelles were evaluated for C–H cyanation of a range of arenes. Our results show that the PMG_100_-*b*-PPG_38_/Mes-Acr-Ph^+^ system operates efficiently in water without the need for organic cosolvents, buffers, or pH adjustment, promoting regioselective cyanation across mono-, di-, and trisubstituted substrates.

The micellar architecture improves catalyst dispersibility, suppresses aggregation, and modulates reactivity by maintaining close proximity of reactants within the confined core. Compared to conventional approaches, this method enhances catalytic performance in water. While TMSCN is hazardous, its use in micellar nanoreactors may limit the rapid release of HCN and improve safety compared to conventional synthetic conditions. However, while solid and highly hydrophobic substrates such as biphenyl and mesitylene showed reduced conversions, likely due to poor solubility or limited compatibility with the micellar core, this method nonetheless represents a rare example of a fully aqueous, polymer-assisted photoredox transformation. The short linear alkyl side chains of the PMG-*b*-PPG block copolypeptoid may hinder effective solubilization and partitioning of bulky or rigid aromatic substrates, thereby reducing their local concentration near the catalytic center. Additionally, the intrinsic redox selectivity of the Mes-Acr-Ph^+^ photocatalyst toward electron-rich arenes further narrows the substrate scope. Despite these limitations, the system demonstrates efficient and selective reactivity with a range of arenes, and the micelles exhibit recyclability, reinforcing their potential for sustainable catalysis. Furthermore, the micelles exhibit recyclability, reinforcing their potential for sustainable catalysis.

This work underscores the value of synthetic micellar scaffolds for overcoming long-standing barriers in photoredox catalysis and opens new avenues for aqueous-phase transformations using nanoscale control of reaction environments. Future efforts will target optimization of micelle architecture and catalyst design to expand reactivity and substrate scope. To enable cosolvent compatibility, we aim to develop micelles with cross-linked or more hydrophobic cores, enhancing structural stability and encapsulation fidelity under demanding conditions. While the observed reactivity trends strongly suggest micelle-assisted solubilization and confinement effects, further scaling via flow reactors and detailed mechanistic investigations (such as excited-state lifetimes and dynamics, local concentration gradients, electron transfer kinetics, and substrate orientation) are ongoing and will be reported in the future.

## Supplementary Material



## Notes

Detailed synthetic procedures, structural characterization of the prepared polymers and arenes using ^13^C and ^1^H NMR spectroscopy, additional experimental results for morphology determination, supplementary TEM and cryo-TEM micrographs of micelles, as well as reactor photographs and process descriptions. (PDF)
