# Supplementary material for: Diblock Copolypeptoid Micelles as Platform for Aqueous Photoredox Cyanation of Arenes
Source: J Am Chem Soc. 2025 Aug 4;147(32):29152–61. doi: 10.1021/jacs.5c07882 (PMC12356584; doi:10.1021/jacs.5c07882)
Supplement: Supplementary file 1 [file ja5c07882_si_004.pdf]

Supporting Information

Diblock Copolypeptoid Micelles as Platform for Aqueous Photoredox Cyanation of Arenes

Afshin Nabiyan* [a], Mitra Esfandiari [a], Jakob Ruickoldt [b], Petra Wendler [b], Nora Kulak [a],

Helmut Schlaad [a]

[a] Institute of Chemistry, University of Potsdam, Karl-Liebknecht-Str. 24-25, 14476 Potsdam, Germany

[b] Institute of Biochemistry and Biology, University of Potsdam, Karl-Liebknecht-Str. 24–25, 14476 Potsdam, Germany

Experimental and Methods

Chemicals

N-Boc-*N*-methylglycine (≥99%, Boc-Sar-OH), mesitylene (99%), 2,6-dimethoxypyridine (99%), phosphorus trichloride (PCl_3, 99%), biphenyl (99.5%), acetic anhydride (99%), glyoxylic acid monohydrate (98%), 1-Aminopropane (99%), Methoxybenzene (99.7 %) and n-heptane (≥99%) were purchased from Sigma-Aldrich and used without further purification. Neopentylamine (>98.0% GC), 1,2,3-trimethoxybenzene (>99%), 1,2-dimethoxybenzene (>99%), and 2-methoxynaphthalene (>98.0% GC) were purchased from TCI. Ethyl acetate (99.8%, anhydrous) was obtained from Acros Chemicals. Dichloromethane (DCM, analytical grade), methanol (analytical grade), 1,3,5-trimethoxybenzene (99%), 2-chloroanisole (98%), 1,3-dimethoxybenzene (99%), and cyclohexane (analytical grade) were purchased from Fisher Scientific. Deuterated NMR solvents were obtained from Deutero GmbH and Eurisotop. Anhydrous *N*-methyl-2-pyrrolidone (NMP, 99.5%) and *N*,*N*-dimethylformamide (DMF, 99%) were purchased from Sigma-Aldrich in septum-sealed bottles containing molecular sieves. 2-Fluoroanisole (99%) and 3,6-bis(1,1-dimethylethyl)-10-phenyl-9-(2,4,6-trimethylphenyl)-acridinium tetrafluoroborate (>97.0%) were purchased from Apollo Scientific Ltd.

Analytical instrumentation and methods

Nuclear magnetic resonance (NMR) spectra were recorded on a Bruker AVANCE NEO 400 MHz spectrometer at room temperature. Samples were prepared in deuterated solvents, specifically $CDCl_3$, DMSO-d_6, methanol-d_4, or DMF-d_7, depending on solubility. Chemical shifts (δ) are reported in parts per million (ppm) and referenced to the residual solvent peaks: $CDCl_3$ (^1H, δ 7.26 ppm), DMSO-d_6 (^1H, δ 2.50 ppm), methanol-d_4 (^1H, δ 3.31 ppm), and DMF-d_7 (^1H, δ 8.03, 2.92, and 2.75 ppm).

Size exclusion chromatography (SEC) with simultaneous UV and differential refractive index (RI) detection was performed using N-methyl-2-pyrrolidone (NMP) containing 0.5 wt% lithium bromide (LiBr) as the eluent. The analysis was conducted at room temperature with a flow rate of 0.5 mL min^{-1}. The separation was achieved using a 300 × 8 mm^2 PSS-GRAM analytical linear column (particle size: 7 μm, molecular weight separation range: 10^2–10^6 Da). Polymer solutions (~0.15 wt%) were carefully prepared by dissolving the sample in the eluent and filtered through 0.45 μm PTFE filters to remove potential particulates before injection. Each sample was injected with a fixed volume of 100 μL to ensure reproducibility. The molar

mass distribution was determined using polystyrene standards (PSS, Mainz, Germany) for calibration.

Dynamic light scattering (DLS) measurements were performed using a Malvern Zetasizer Nano ZS at room temperature (ca. 25 °C) to determine the hydrodynamic diameter and size distribution of micelles or polymer solutions. Samples (0.1 wt%) were carefully filtered through 2.0 µm PTFE filters to remove dust and aggregates before measurement. The filtered solutions were then transferred into 1.5 mL disposable cuvettes.

UV–visible absorption spectra of Mes-Acr-Ph^+ and polymeric micelles loaded with Mes-Acr-Ph^+ were recorded using a PerkinElmer Lambda 2 spectrometer at room temperature (ca. 25 °C). Mes-Acr-Ph^+ was measured in acetonitrile, while the polymeric micelles were analyzed in an aqueous solution, ensuring appropriate solubility conditions. Spectra were collected over the 200–800 nm wavelength range with a 1 nm spectral resolution, using quartz cuvettes.

Transmission Electron Microscopy (TEM): All samples were imaged using a JEOL JEM 1011 transmission electron microscope (JEOL, Akishima, Tokyo, Japan) equipped with an Olympus MegaView G2 camera at an acceleration voltage of 80 kV. For TEM imaging, copper grids with a 1 nm carbon layer on top of a 10 nm Formvar film (EFCF400-Cu-50, Science Services GmbH, Unterhachinger Straße 75, Munich, Germany) were used. To enhance sample adhesion and remove organic contaminants, the TEM grids were plasma-treated for 15 s using a Diener Electronic Zepto plasma cleaner before sample deposition. To characterize the micelles, 5 µL of the micellar solution was pipetted onto the TEM grid and incubated for 2–3 minutes before imaging.

Cryogenic transmission electron microscopy (cryo-TEM) analysis was performed on a Thermo Fisher Talos F200C G2 field emission gun TEM operating at 200 keV. 4 µL of the micellar solution was applied to freshly glow discharged Quantifoil R1.3/1.2 300-mesh holey carbon grids. The sample was incubated for 45 s at 10 °C and 80% humidity before blotting for 4 sec and plunge freezing into liquid ethane on a Leica EM GP2 plunge freezer. Images were recorded with EPU (Thermo Scientific) on a 4k x 4k Falcon III camera at a magnification of 57,000x pixel (pixel size 0.254nm).

Gas chromatography–mass spectrometry (GC-MS) Analysis was performed using an HP 6890 Series Gas Chromatograph (Hewlett-Packard) coupled to an Agilent Technologies 5973 Network Mass Selective Detector (MSD). Sample separation was carried out on the GC

system, followed by detection using electron ionization (EI) and time-of-flight (TOF) mass spectrometry. Both low- and high-resolution mass spectra were obtained under EI/TOF conditions. Data acquisition and processing were conducted using GCMS5973 Data Analysis software, and further interpretation was supported by Enhance Data Analysis tools.

Electrochemical Measurements. Cyclic voltammetry (CV) experiments were performed using a µStat 400 Bipotentiostat/Galvanostat (Metrohm DropSens) in conjunction with DropView 8400 software for data acquisition and analysis. Measurements were conducted in an aqueous solution of 100 mM KCl as the supporting electrolyte. The electrochemical cell was based on a screen-printed carbon electrode (SPCE, Metrohm DropSens 11L), consisting of a carbon working electrode, a carbon counter electrode, and an integrated Ag/AgCl reference electrode, all printed on a ceramic substrate.

Cyclic voltammograms were recorded over a potential range of −0.1 V to +0.6 V (vs. Ag/AgCl) at scan rates varying from 25 $mV{\cdot}s^{-1}$ to 1000 $mV{\cdot}s^{-1}$. All measurements were initiated at a starting potential of +0.1 V, with the first scan conducted in the anodic (positive) direction. The concentration of the analyte solutions was maintained at 3 $mg{\cdot}mL^{-1}$ for all measurements.

Monomer syntheses

N-Methylglycine (sarcosine) *N*-carboxyanhydride (Sar NCA)

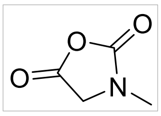

Sar NCA was synthesized in a one-step reaction following previously reported methods.[1–3] Briefly, *N*-Boc-*N*-methylglycine (Boc-Sar-OH) (5.63 g, 30 mmol) was placed in a three-neck round-bottom flask equipped with a condenser and maintained under an argon atmosphere. Dry DCM (150 mL) was added to dissolve Boc-Sar-OH completely, and the mixture was stirred at 0 °C for 30 min. A separate solution of PCl_3 (2.2 mL, 25 mmol) in 10 mL of dry DCM was prepared and added dropwise to the reaction mixture while continuously bubbling argon. The reaction was allowed to proceed for 3 h at 0 °C, leading to the formation of Sar NCA. Upon completion, the solvent and excess volatiles were removed under reduced pressure using a rotary evaporator, yielding a yellowish oil as the crude product. The crude oil was dissolved in dry DCM, filtered to remove insoluble impurities, and further purified by sublimation at 50 °C under high vacuum (6.4×10^{-5} mbar), affording Sar NCA as colorless crystals with a final yield of 2.27 g (70%). ^1H NMR (400 MHz, $CDCl_3$): δ 4.10 (s, 2H, CH_2), 3.00 (s, 3H, CH_3).

N-n-Propylglycine NCA (PG NCA)

N-n-Propylglycine NCA was synthesis in three steps according to previous reports.[1,3]

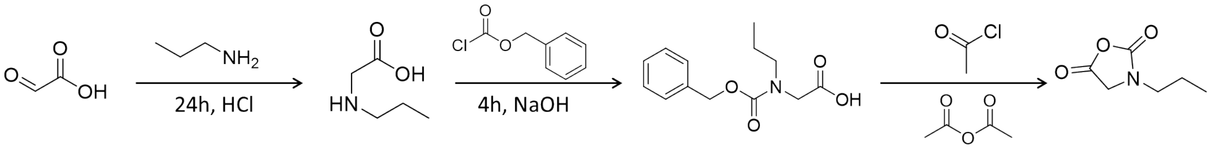

(i) *N*-n-Propylglycine hydrochloride

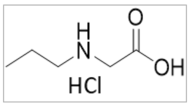

Glyoxylic acid monohydrate (13.3 g, 405 mmol) was dissolved in 500 mL of dry DCM and mixed with n-propylamine (10.0 mL, 170.2 mmol) at room temperature under continuous stirring. The reaction was allowed to proceed for 24 h, after which volatile solvents were removed under reduced pressure via rotary evaporation. The resulting intermediate was dissolved in 500 mL of 1 M HCl and refluxed for 24 h to ensure complete formation of *N*-n-propylglycine hydrochloride. The aqueous medium was removed by rotary evaporation, and the crude solid was purified by recrystallization using a methanol /diethyl ether mixture (1.5:1 v/v).

(ii) *N*-(Benzyloxycarbonyl)-*N*-n-propylglycine

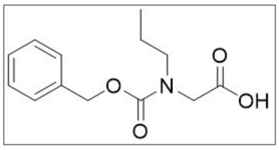

In the next step, a suspension of N-n-propylglycine hydrochloride (8.8 g, 58 mmol) was prepared in 100 mL of toluene and cooled in an ice bath. Separately, NaOH (7.0 g, 177 mmol) was dissolved in 80 mL of deionized water and added to the suspension, followed by the dropwise addition of benzyl chloroformate (9.6 g, 57 mmol) with continuous stirring at room temperature. The reaction mixture was stirred for 4 h, then allowed to separate into organic and aqueous layers. The aqueous layer was extracted, its pH adjusted to 1–2 using concentrated HCl, and then recombined with the organic phase in a separatory funnel. The organic layer was extracted with ethyl acetate (3 × 300 mL), dried over anhydrous $MgSO_4$, filtered, and concentrated under reduced pressure to yield *N*-(benzyloxycarbonyl)-*N*-n-propylglycine as a viscous yellow oil (11.0 g, 85%).

(iii) N-n-Propylglycine NCA

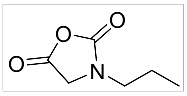

In the final step, *N*-(benzyloxycarbonyl)-*N*-n-propylglycine (13.5 g, 54 mmol) was combined with acetic anhydride (11.0 g, 198 mmol) and acetyl chloride (8.6 g, 110 mmol) in a two-neck round-bottom flask fitted with a water-cooled condenser under an argon atmosphere. The reaction mixture was refluxed for 6 h, after which the

volatile organics were removed by rotary evaporation, yielding a crude yellow oil. The product was purified via fractional distillation under vacuum (0.04 mbar) with a heated condenser (~120 °C), affording *N*-n-propylglycine NCA as a colorless oil (2 g, 40%). ^{1}H NMR (400 MHz, $CDCl_3$,): δ (ppm) 4.14 (s, 2H, CO-CH_2-N), 3.30 (t, 2H, NCH_2), 1.60 (m, 2H, -CH_2-), 0.95 (t, 3H, -CH_3).

Polymer synthesis

Poly(*N*-methylglycine) (PMG)

Sar-NCA (500 mg, 4.3 mmol) was weighed into a reaction vessel and dissolved in 3 mL of dry DMF under an argon atmosphere. Once fully dissolved, neopentylamine (5 µL, 0.04 mmol) was dissolved in 1 mL of dry DMF and added to the Sar-NCA/DMF solution under argon. The reaction mixture was stirred at room temperature under constant reduced pressure (60 mbar) for 24 h. After completion, the reaction mixture was precipitated into 150 mL of diethyl ether, and the isolated PMG was dried under reduced pressure. The precipitation step was repeated twice, after which the final compound was dissolved in water and freeze-dried. ^{1}H NMR (400 MHz, DMSO-d_6): **Figure S1** left, degree of polymerization, n = 100 (calculated based on methylene signal *2*), M_n 7.2 kg/mol. SEC (NMP): **Figure S1** right, M_n^{app} 8.0 kg/mol, $Đ$ 1.2 (polystyrene calibration).

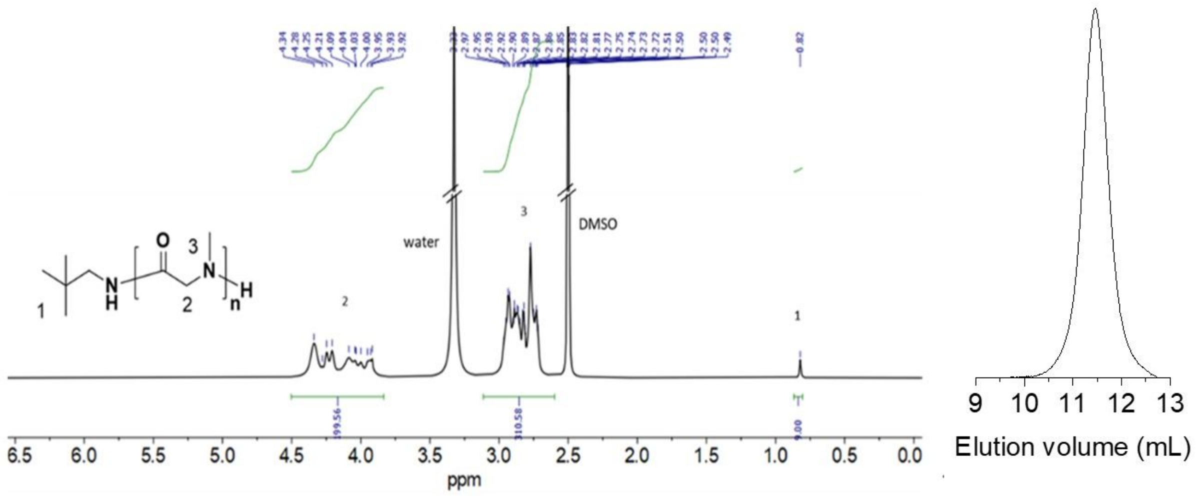

Figure S1. ^{1}H NMR (400 MHz, DMSO-d_6) spectrum (left) and SEC-RI trace (eluent: NMP) (right) of PMG.

Poly(*N*-methylglycine)-*block*-poly(*N*-n-propylglycine) (PMG-*b*-PPG)

200 mg (0.027 mmol) of the PMG_{100} macroinitiator was dissolved in 1 mL of dry DMF under an argon atmosphere. Separately, a solution of PG-NCA was prepared by dissolving 140 mg (0.98 mmol) of the monomer in 1 mL of dry DMF under argon. The reaction mixture was

stirred at room temperature under reduced pressure (60 mbar) for 48 h to facilitate polymerization and the removal of the CO_2 byproduct. Upon completion, the reaction mixture was precipitated dropwise into cold diethyl ether. The precipitated polymer was purified by dialysis against a mixture of deionized water and methanol using a 3.5 kDa molecular weight cut-off membrane to remove residual monomers and solvents, yielding 80% of the final product. ^1H NMR (400 MHz, DMSO-d_6): **Figure S2** left, mole fraction PG = 0.27 (calculated based on PG methylene signal *5*), degree of polymerization, m = 38, M_n 10.9 kg/mol. SEC (NMP): **Figure S2** right, M_n^{app} 11.0 kg/mol, $Đ$ 1.1 (polystyrene calibration).

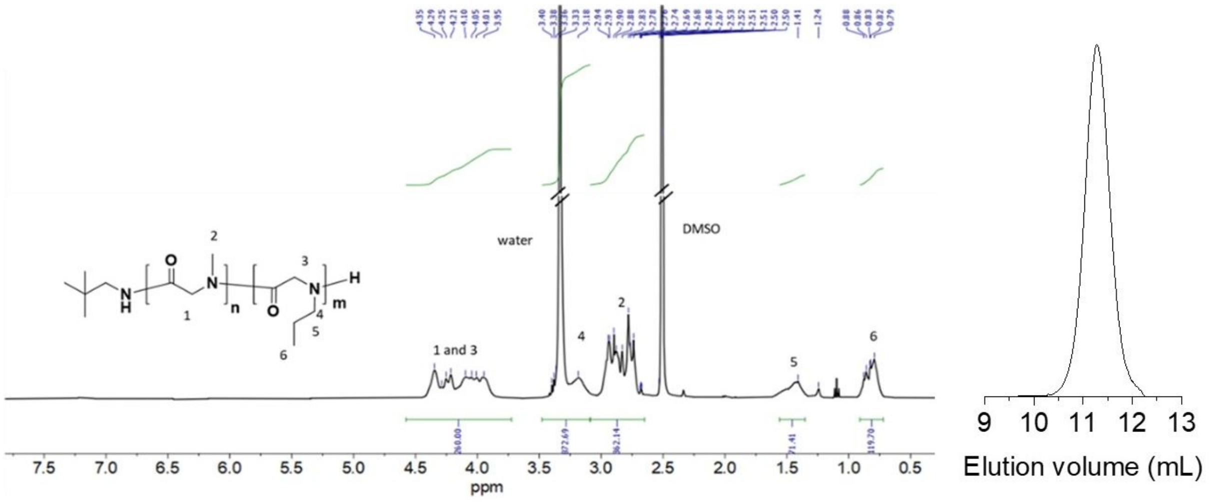

Figure S2. ^1H NMR (400 MHz, DMSO-d_6) spectrum (left) and SEC-RI trace (eluent: NMP) (right) of PMG-*b*-PPG.

Formation of PMG_{100}-*b*-PPG_{38} Micelles

A 5 mg sample of the PMG_{100}-*b*-PPG_{38} was dissolved in 2 mL of methanol. Water (4 mL) was then added dropwise to the polymer/methanol solution, leading to the formation of a slightly turbid mixture, which was stirred for 1 h. Subsequently, an additional 5 mL of water was rapidly added, and the dispersion was stirred overnight. To remove methanol, the mixture was dialyzed against water (MWCO 8 kDa) for at least 24 h, with a minimum of eight water changes.

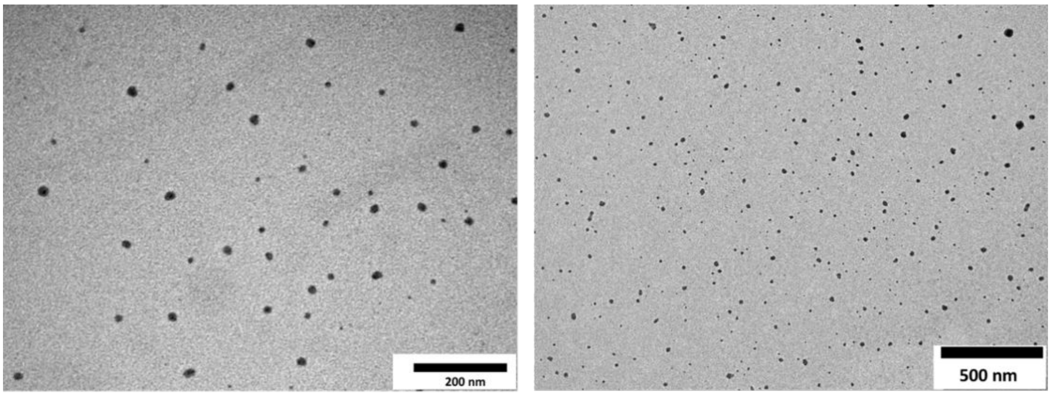

Figure S3. TEM micrographs of PMG_{100}-*b*-PPG_{38} micelles drop-casted from a 1 mg/mL aqueous solution.

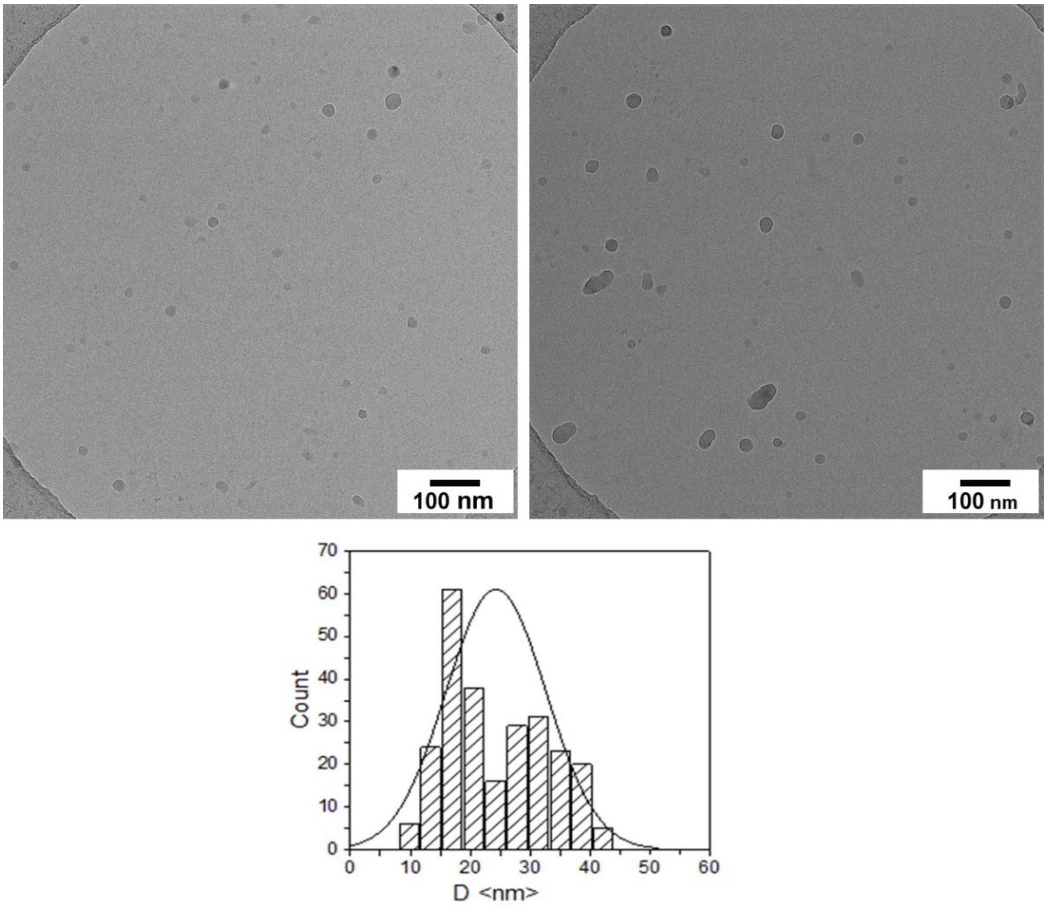

Figure S4. Cryo-TEM micrographs of PMG_{100}-*b*-PPG_{38} micelles in a 3 mg/mL aqueous solution. Bottom: Histogram of the particle size distribution.

Formation of PMG-*b*-PPG/Mes-Acr-Ph^+ Micelles

A 5 mg sample of the PMG_{100}-*b*-PPG_{38} and 5 mg (0.008 mmol) of Mes-Acr-Ph^+ dye were simultaneously dissolved in 2 mL of methanol under continuous stirring to ensure complete dissolution. To promote micelle formation, 4 mL of Milli-Q water was added dropwise under constant stirring. This process resulted in the formation of a yellow, turbid dispersion, indicating the successful incorporation of Mes-Acr-Ph^+ into the micellar structures. The dispersion was stirred for 1 h to allow equilibration. Subsequently, an additional 5 mL of water was rapidly added to enhance micelle stabilization and complete the self-assembly process. The resulting suspension was stirred overnight at room temperature under an argon flow to ensure uniform micelle formation and partial removal of methanol. To remove further the residual methanol and further purify the micellar dispersion, the mixture was subjected to dialysis against Milli-Q water using a membrane with a molecular weight cut-off of 8 kDa. Dialysis was performed for at least 5 h, with a minimum of ten water changes, ensuring maximum removal of methanol.

A 4 M phosphate buffer at pH 9 was prepared following a previously reported method.[4] Briefly, 0.39 g (2.9 mmol) of potassium phosphate monobasic (KH_2PO_4) and 69.16 g (397 mmol) of potassium phosphate dibasic (K_2HPO_4) were accurately weighed and dissolved in 100 mL of distilled water.

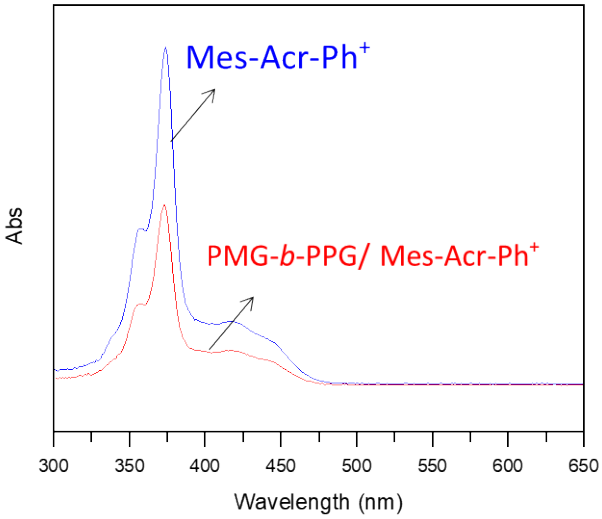

Figure S5. UV-visible absorption spectra of Mes-Acr-Ph+ in acetonitrile (0.010 mg/mL) (blue line) and PMG_{100}-*b*-PPG_{38}/Mes-Acr-Ph+ in water (0.10 mg/mL) (red line).

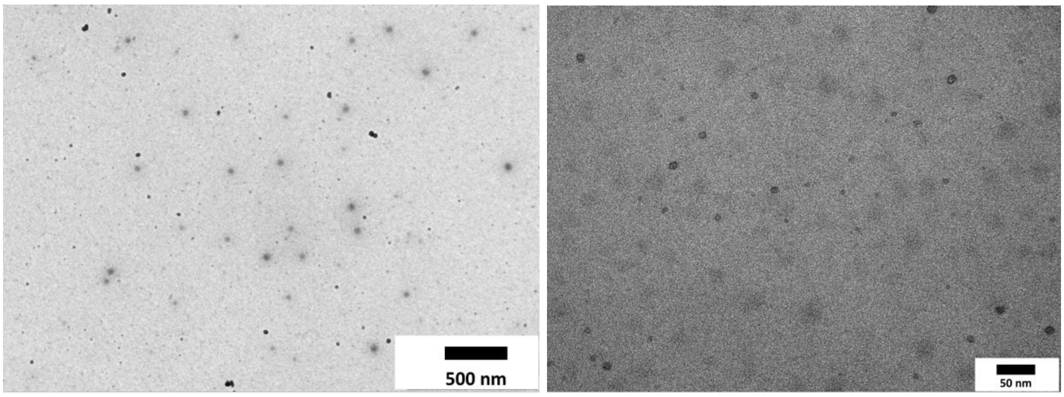

Figure S6. TEM micrographs of PMG_{100}-*b*-PPG_{38}/Mes-Acr-Ph+ micelles drop-casted from a 1 mg/mL aqueous solution.

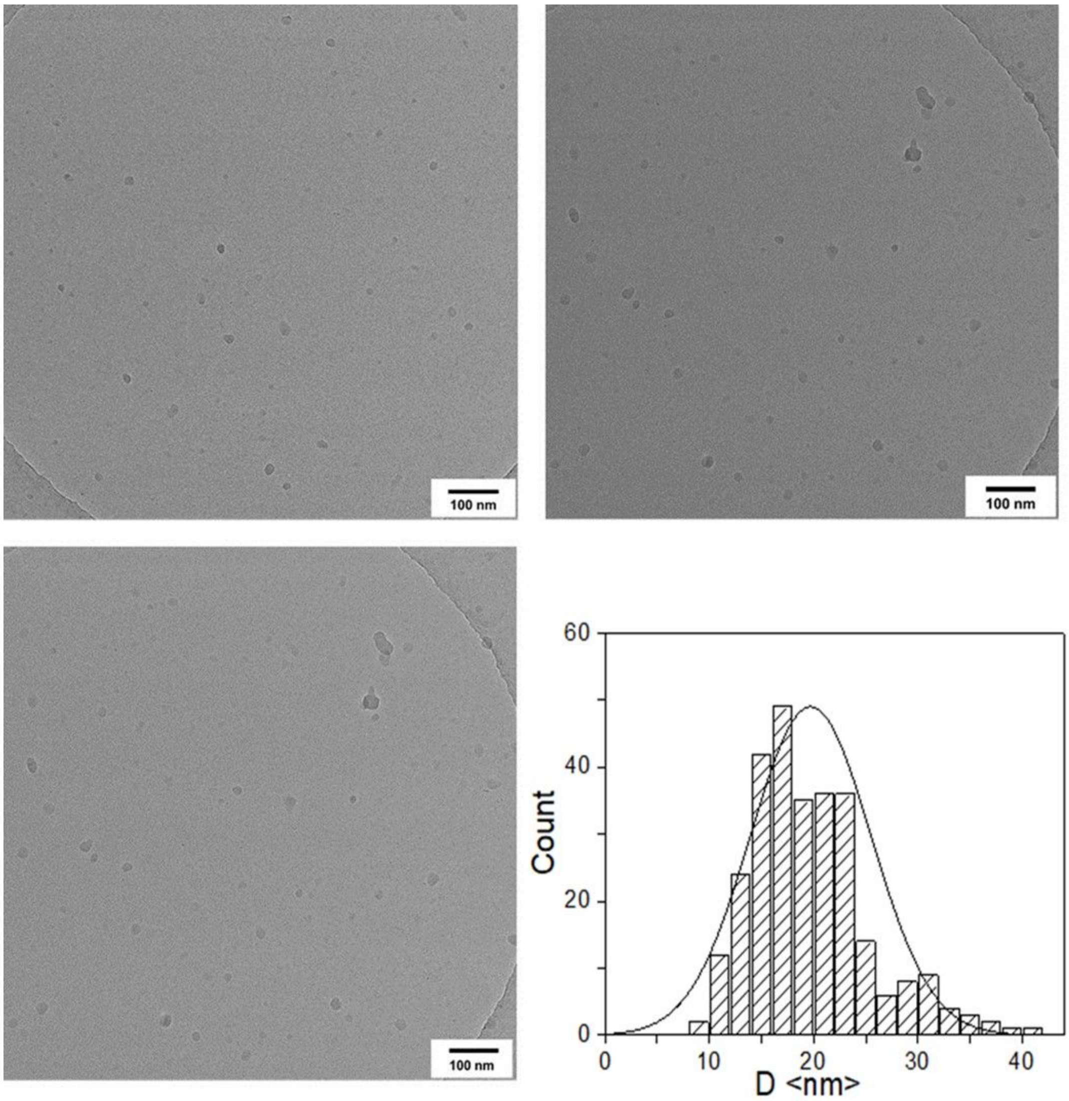

Figure S7. Cryo-TEM micrographs of PMG_{100}-*b*-PPG_{30}/Mes-Acr-Ph^{+} micelles in a 4 mg/mL aqueous solution. Bottom: Histogram of the particle size distribution.

Light-driven cyanation by micelles

Photoreactor: Photocatalytic experiments were carried out in a custom-made, 3D printed photoreactor equipped with a ventilating fan and an Opulent Americas LED (SST-10-B-B90-Q450) with a maximum wavelength of 450 nm (with ± 50 nm; 590mW; 350 mA; 3 V).[5,6]

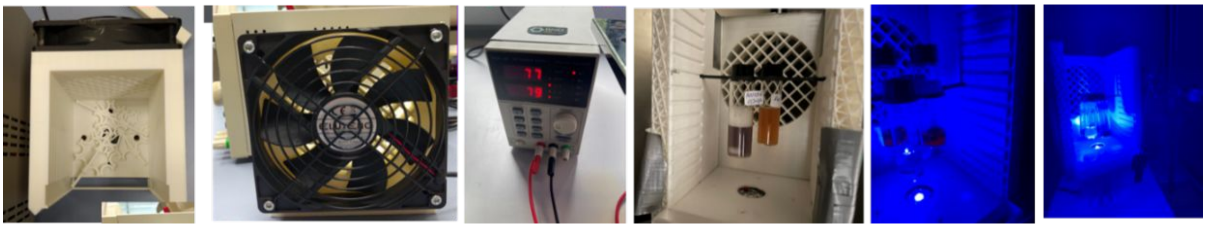

The photocatalytic arene cyanation was conducted in a 5 or 10 mL Pyrex flask sealed with a silicone rubber septum at ambient temperature and atmospheric pressure. A 450 nm LED light source, positioned 2 cm from the flask in a 3D-printed holder, provided irradiation. In typically example, a micellar photocatalyst solution (0.1 mg/mL) was loaded into the reaction vial, followed by the addition of the arene substrate to achieve a concentration of 0.03 mM. The mixture was stirred for 30 min, after which trimethylsilyl cyanide TMSCN was added to a final concentration of 0.07 mM. High-purity oxygen gas (99.99%) was bubbled through the solution for 10 minutes to ensure proper oxygenation. The reaction mixture was then irradiated for the desired duration. After completion, the organic products were extracted three times using ethyl acetate and n-hexane and purified by silica gel column chromatography to obtain the aryl nitrile product. Attempts to increase substrate concentrations beyond 0.1 mM resulted in solution discoloration and reduced reactivity, consistent with micelle destabilization and photocatalyst decomposition. These effects, along with reduced light penetration in larger batch volumes, currently limit substrate loading. To overcome these constraints, future studies will adapt the system to continuous-flow conditions to enable improved scalability and higher concentration operation.

Table S1. Optimization of Reaction Conditions for the Cyanation of 2,6-DMP With TMSCN Using PMG_{100}-*b*-PPG_{38}/Mes-Acr-Ph^{+} Micelles in Water.

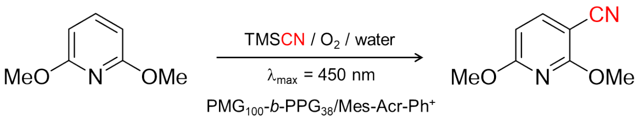

Entry [a]	Atm [b]	pH	TMSCN/2,6-DMP [f]	Conv. [%] [g]

1	O_2	8-9 [c]	2.6	> 95
2	O_2	~6[d]	no TMSCN	0
3	O_2	~6[d]	1.3	69
4	O_2	8-9 [c]	1.3	80
5	O_2	9-10 [c]	2.6	> 95
6	O_2	11-12 [c]	2.6	> 95
7	O_2	8-9 [e]	2.6	60

[a] Reactions were carried out at PMG_{100}-*b*-PPG_{38}/Mes-Acr-Ph^+ (0.2 mg/mL), under irradiation with one LED lamp (λ_{max} = 450 nm) for 24 h, [b] O_2 purged for 15 min, [c] pH was adjusted by saturated NaOH solution, [d] Milli-Q water was used directly as solvent without pH adjustment, [e] pH was adjusted by saturated NaOH and 4 M potassium phosphate buffer solution, [f] molar ratio TMSCN to 2,6-DMP (0.03 mM) used in cyanation reactions, and [g] conversion was obtained from the crude reaction mixture by ^{1}H NMR spectroscopy.

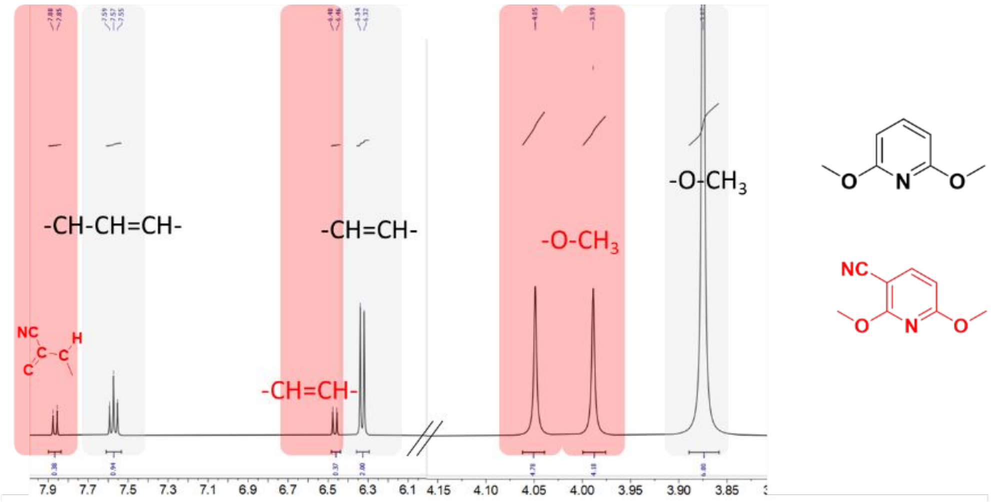

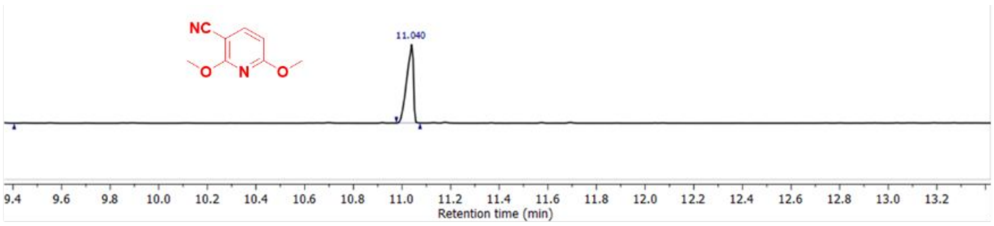
NC
O
N
O
11.040
9.4
9.6
9.8
10.0
10.2
10.4
10.6
10.8
11.0
11.2
11.4
11.6
11.8
12.0
12.2
12.4
12.6
12.8
13.0
13.2
Retention time (min)

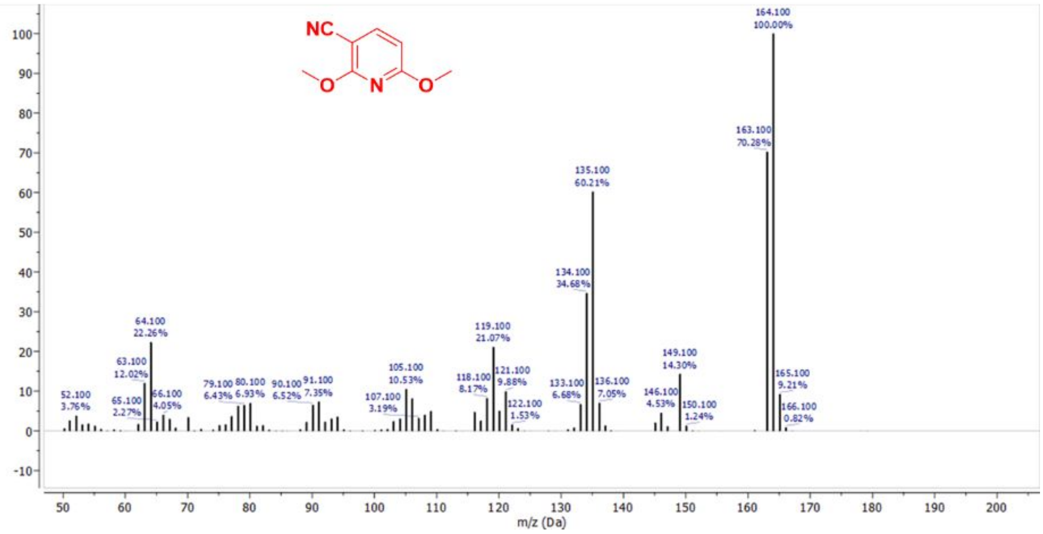
NC
O
N
O
164.100 100.00%
163.100 70.28%
135.100 60.21%
134.100 34.68%
64.100 22.26%
119.100 21.07%
149.100 14.30%
63.100 12.02%
105.100 10.53%
121.100 9.88%
165.100 9.21%
118.100 8.17%
91.100 7.35%
136.100 7.05%
80.100 6.93%
133.100 6.68%
90.100 6.52%
79.100 6.43%
146.100 4.53%
66.100 4.05%
52.100 3.76%
107.100 3.19%
65.100 2.27%
122.100 1.53%
150.100 1.24%
166.100 0.82%
100
90
80
70
60
50
40
30
20
10
0
-10
50
60
70
80
90
100
110
120
130
140
150
160
170
180
190
200
m/z (Da)

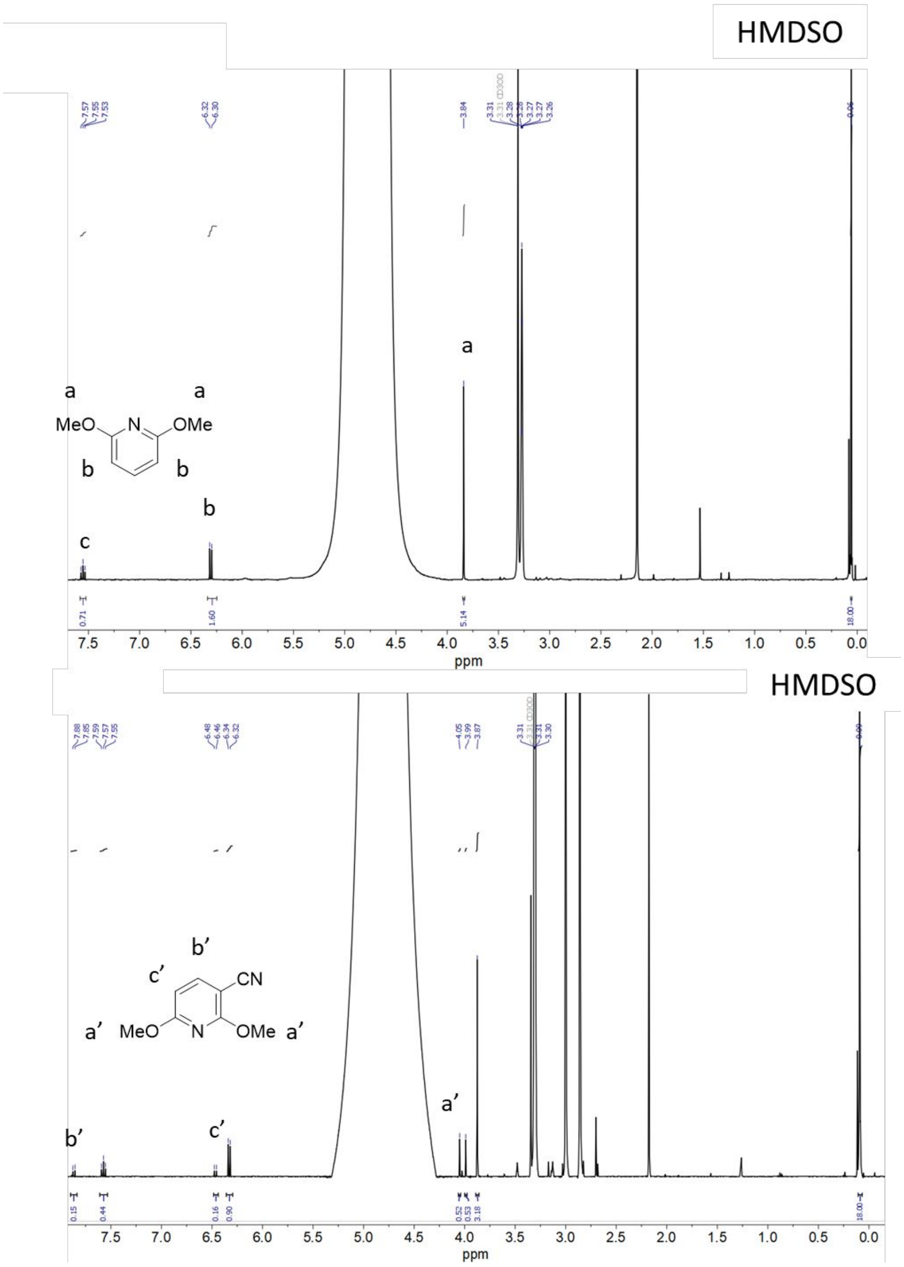

Figure S8. Top: ^{1}H NMR (400 MHz, CD_3OD) (3.8-8.0 ppm) and GC-MS spectra of the reaction mixture for Table S1, entry 4, reaction time 16 h. Bottom: ^{1}H NMR spectra of the crude reaction mixture before irradiation and after 16 h of irradiation for determination of conversion, referencing to the signal of the HMDSO internal standard.

NMR and GC-MS analyses corresponding to Table 1, entry 5 and Table 2, entries 1-7, which summarizes the scope of the arene cyanation reaction carried out in water using PMG_{100}-*b*-PPG_{38}/Mes-Acr-Ph^+ micelles. In representative cases, isolated yields were found to be in close agreement with NMR-based conversions, supporting the use of conversion as a reliable proxy for yield in this screening context.

Analysis of reaction products referring to Table 1, entry 5: 2,6-Dimethoxynicotinonitrile: The

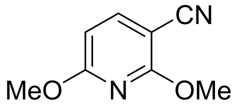

product was isolated as a white solid by column chromatography using 0–6% ethyl acetate in hexane (EtOAc/Hex) as the eluent. The analytical data obtained matched the values reported in the literature.[4,7] ^{1}H NMR (400 MHz, $CDCl_3$) δ 7.70 (d, *J* = 8.3 Hz, 1H), 6.36 (d, *J* = 8.3 Hz, 1H), 4.04 (s, 3H), 3.98 (s, 3H). ^{13}C NMR (101 MHz, $CDCl_3$) δ 165.5, 164.7, 144.1, 116.0, 102.6, 86.7, 54.2, 54.0. *m/z* calculated for [M^+]: 164.06; found: 164.1.

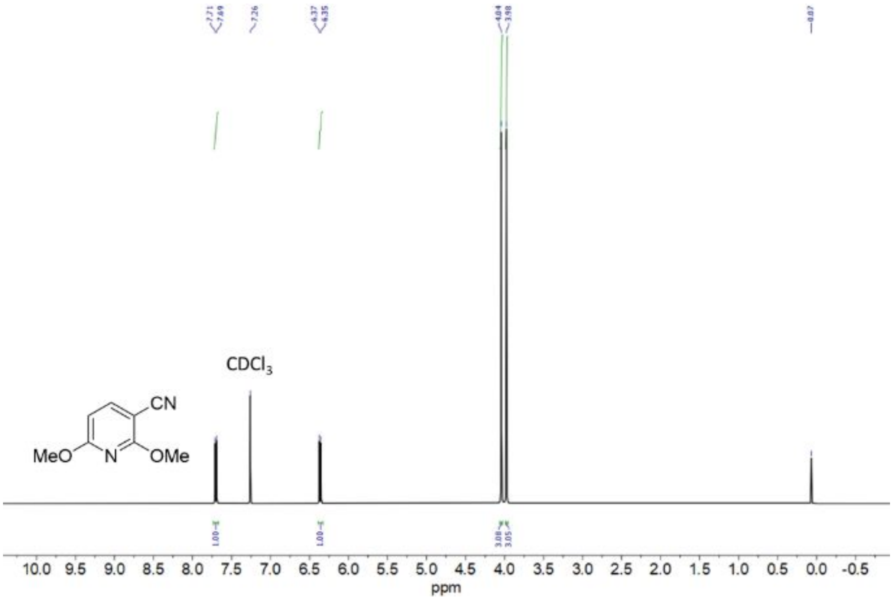

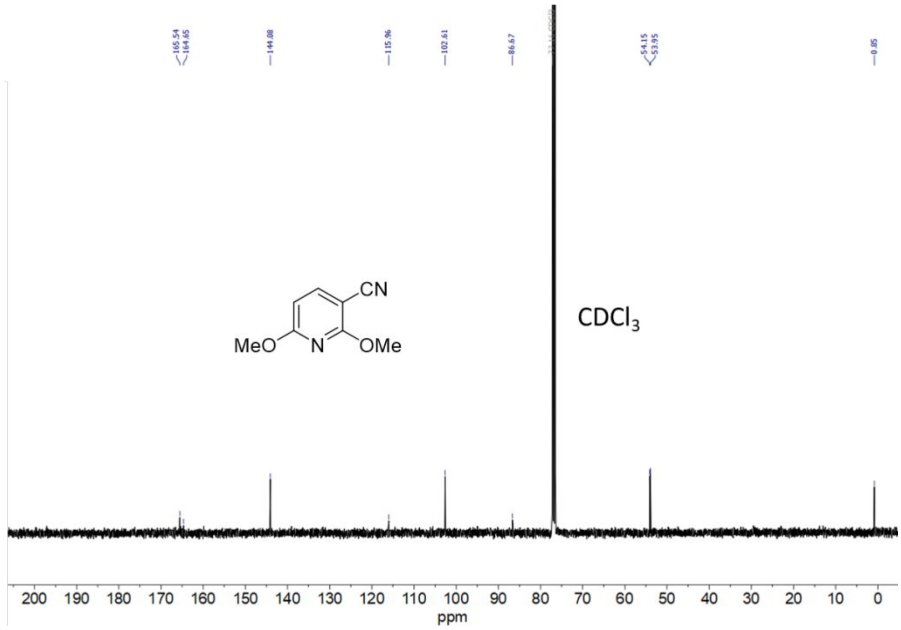

Figure S9. ^{1}H NMR (400 MHz, $CDCl_3$) and ^{13}C NMR (101 MHz, $CDCl_3$) spectra of the cyanation product corresponding to Table 1, entry 5.

Analysis of reaction products referring to Table 2, entry 1: Mix of 2-methoxybenzonitrile and

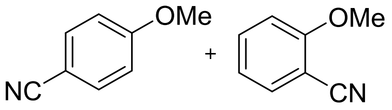

4-methoxy-benzonitrile: The product was isolated as a mixture (white solid) by column chromatography using 10-20 % ethyl acetate in hexane (EtOAc/Hex) as the eluent. The analytical data obtained matched the values reported in the literature.[11,12] ^{1}H NMR (400 MHz, $CDCl_3$) ^{1}H NMR (600 MHz, CDCl3) δ 7.58 (d, *J* = 9.0 Hz, 2H), 7.53-7.52 (m, 1H), 7.03-6.98 (m, 1H), 6.94-6.96 (d, *J* = 8.7 Hz, 2H), 3.94 (s, 1.5H), 3.89 (s, 3H). *m/z* calculated for [M$^+$]: 133.05; found: 133.1.

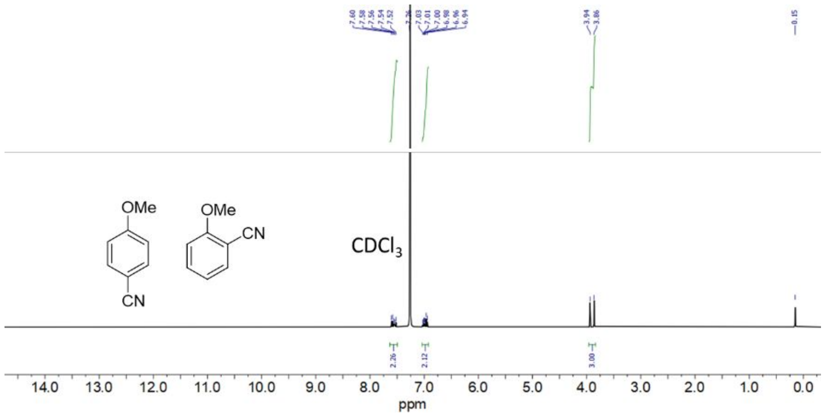

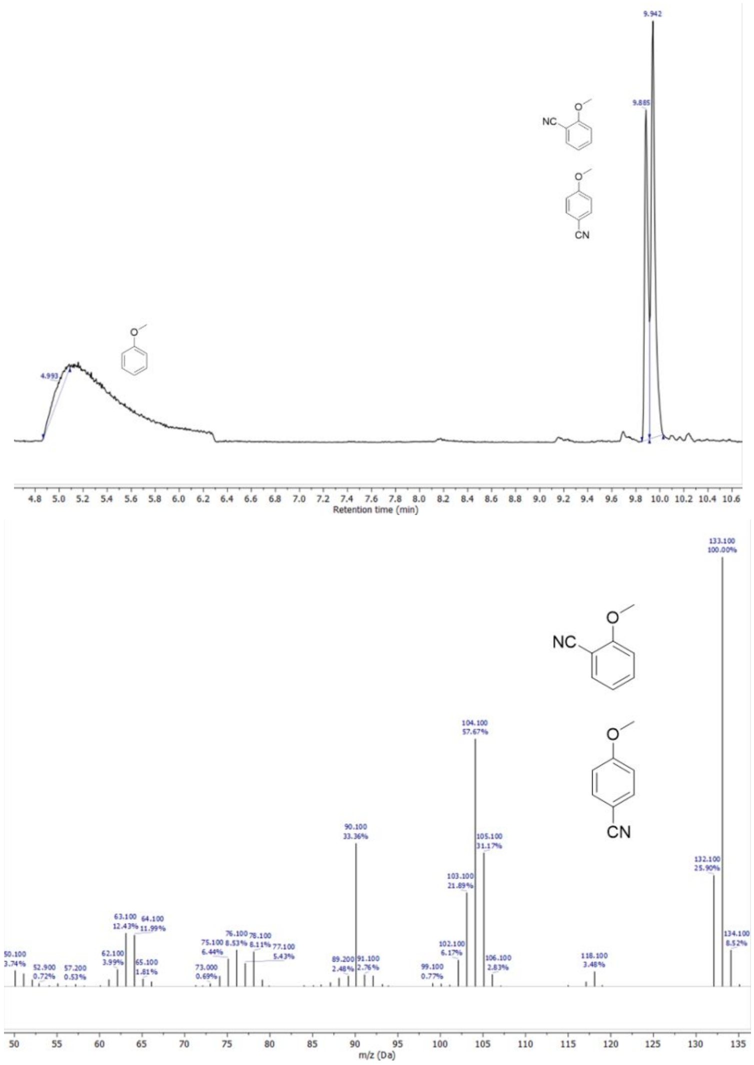

Figure S10. ^{1}H NMR (400 MHz, $CDCl_3$) and GC-MS spectra of the product from Table 2, entry 1.

Analysis of reaction products referring to Table 2, entry 2: Mix of 2-methoxybenzonitrile and

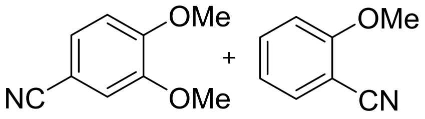

3,4-di-methoxybenzonitrile: The product was isolated as a mixture by column chromatography using 10-30 % ethyl acetate in hexane (EtOAc/Hex) as the eluent. The analytical data obtained matched the values reported in the literature.[4,11] ^{1}H NMR (400 MHz, $CDCl_3$) 7.26 (t, *J* = 8.0 Hz, 1H), 7.11 (s, 1H), 6.97 (d, *J* = 8.5 Hz, 1H), 3.4.06 (s, 3H), 3.92 (s, 3H). 1H NMR (400 MHz, $CDCl_3$) 7.51-7.52 (m, 1H), 7.02-6.74 (m, 2H), 3.70 (s, 3H). *m/z* calculated for [M^+]: 163.06 and 133.05; found: 163.1.and 133.1.

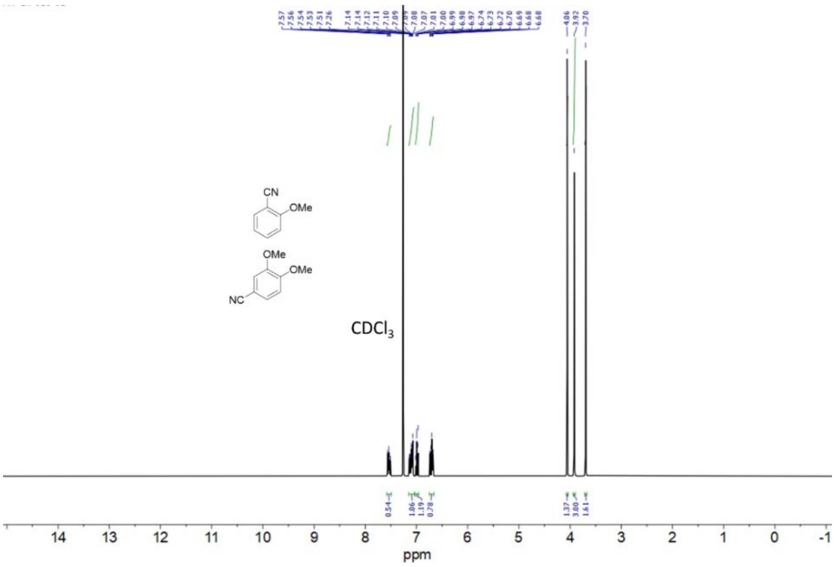
CN
OMe
OMe
OMe
NC
CDCl3
ppm

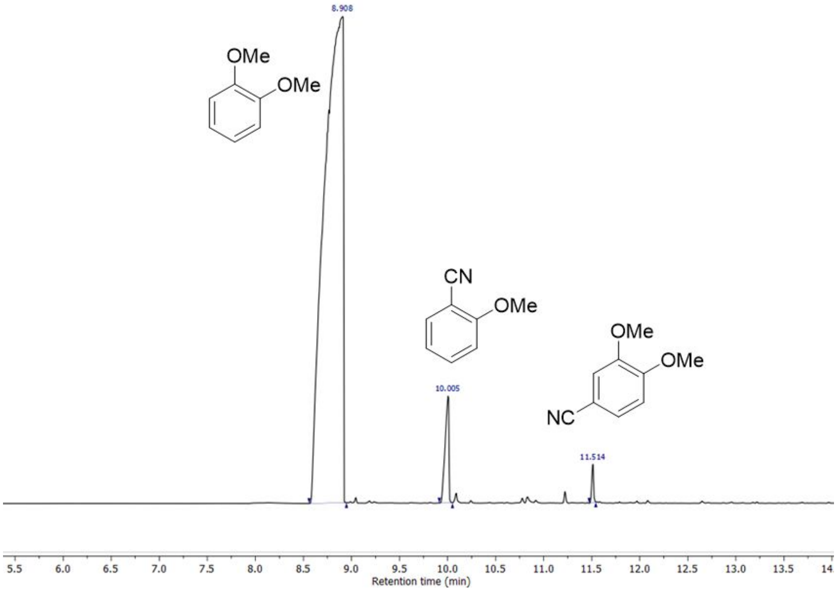
8.908
OMe
OMe
CN
OMe
10.005
OMe
OMe
NC
11.514
Retention time (min)

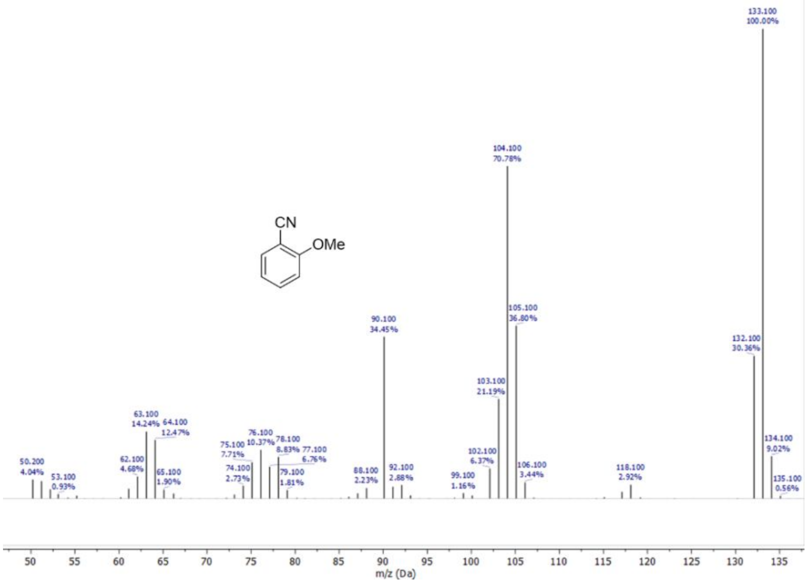
133.100
100.00%
104.100
70.78%
CN
OMe
105.100
36.80%
90.100
34.45%
132.100
30.36%
103.100
21.19%
63.100
14.24%
64.100
12.47%
76.100
10.37%
78.100
8.83%
75.100
7.71%
77.100
6.76%
134.100
9.02%
62.100
4.68%
102.100
6.37%
50.200
4.04%
53.100
0.93%
65.100
1.90%
74.100
2.73%
79.100
1.81%
88.100
2.23%
92.100
2.88%
99.100
1.16%
106.100
3.44%
118.100
2.92%
135.100
0.56%
m/z (Da)

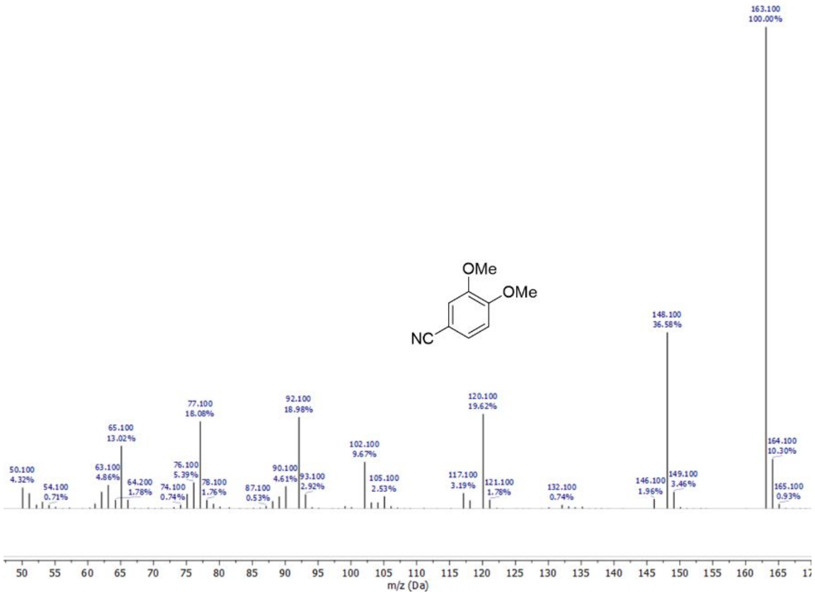

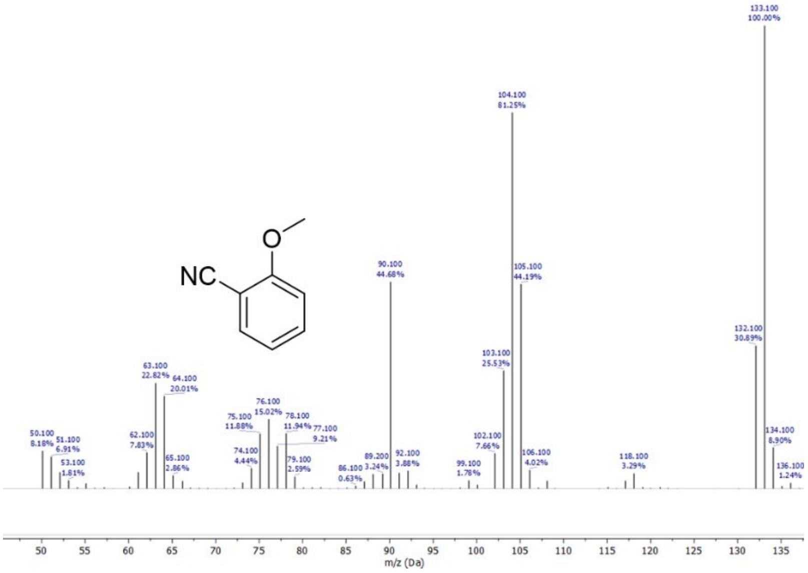

Figure S11. ^{1}H NMR (400 MHz, $CDCl_3$) and GC-MS spectra of the product from Table 2, entry 2.

Analysis of reaction products referring to Table 2, entry 3: 2,4-Dimethoxybenzonitrile: The

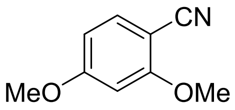

product was isolated as a reddish solid by column chromatography using 30% ethyl acetate in hexane (EtOAc/Hex) as the eluent. The analytical data obtained matched the values reported in the literature.[4,8] ^{1}H NMR (400 MHz, $CDCl_3$) δ 7.48 (d, *J* = 8.6 Hz, 1H6.51 (dd, *J* = 8.6, 2.3 Hz, 1H), 6.45 (d, *J* = 2.3 Hz, 1H), 3.90 (s, 6H). ^{13}C NMR (101 MHz, $CDCl_3$) δ 165.1, 163.3, 135.4, 117.4, 106.2, 99.0, 94.6, 56.4, 56.2. *m/z* calculated for [M^+]: 163.06; found: 163.1.

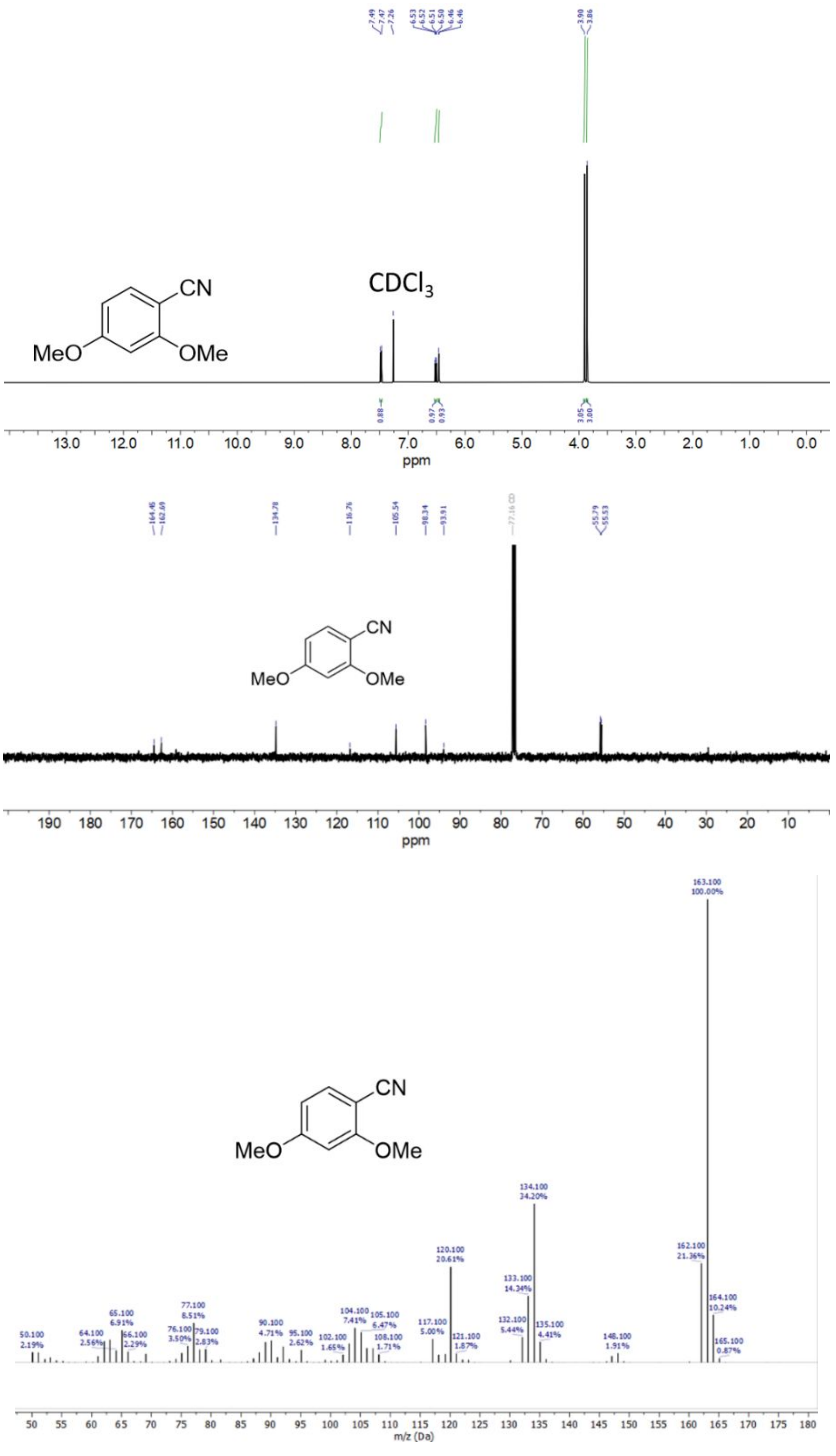

Figure S12. ^{1}H NMR (400 MHz, $CDCl_3$), ^{13}C NMR (101 MHz, $CDCl_3$), and GC-MS spectra of the product from Table 2, entry 3.

Analysis of reaction products referring to Table 2, entry 4: 2,4,6-Trimethoxybenzonitrile: The

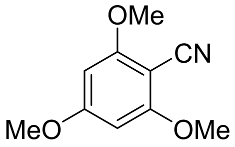

product was isolated as a light-yellow solid by column chromatography using 30% ethyl acetate in hexane (EtOAc/Hex) as the eluent. The analytical data obtained matched the values reported in the literature.[8,9] ^{1}H NMR (400 MHz, $CDCl_3$) δ 6.06 (s, 2H), 3.87 (s, 6H), 3.85 (s, 3H). ^{13}C NMR (101 MHz,

CDCl$_3$): 165.3, 163.8, 114.6, 90.3, 84.1, 56.1, 55.7,55.3. *m/z* calculated for [M$^+$]: 193.07; found: 193.2.

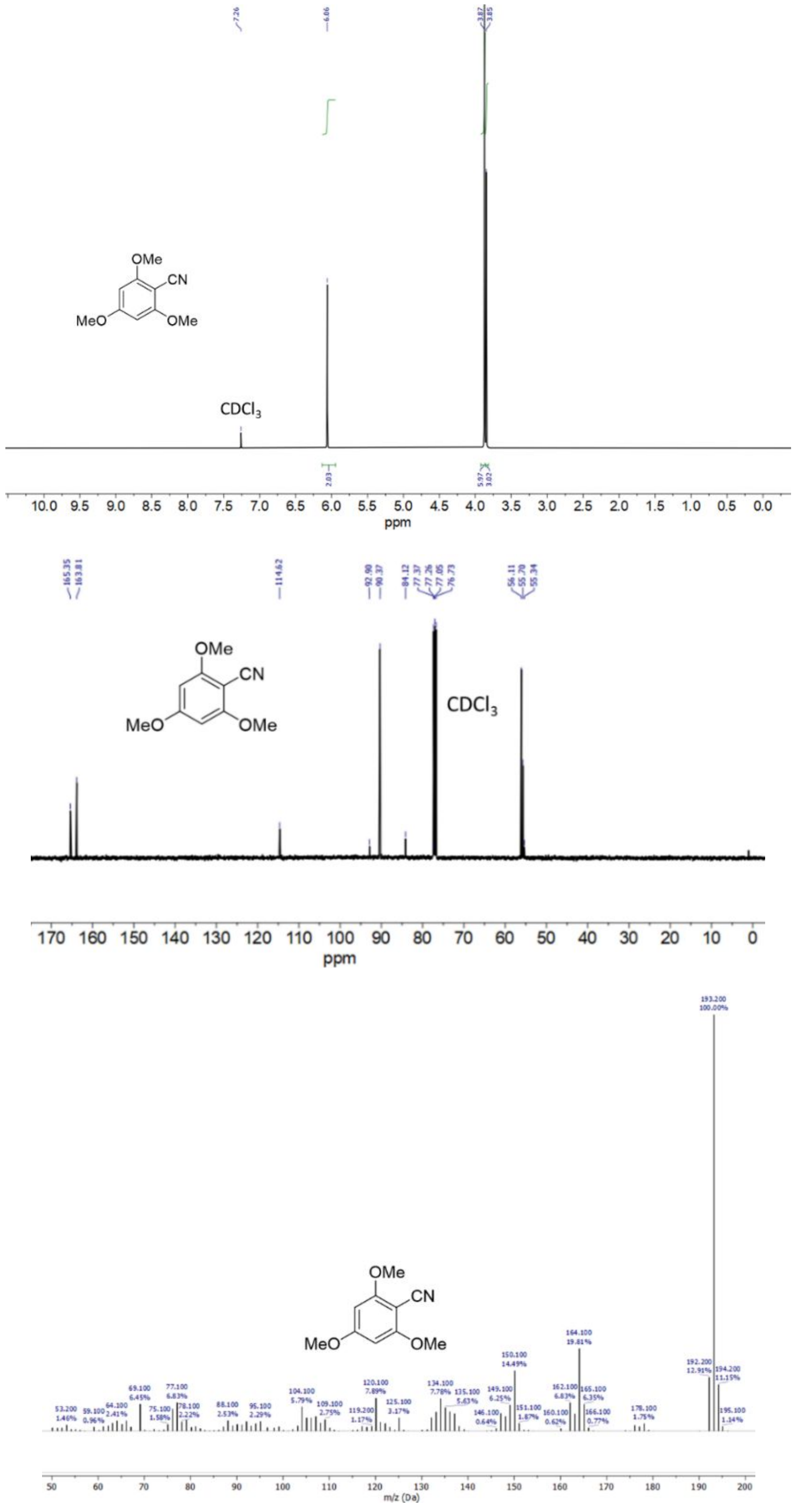

Figure S13. ^{1}H NMR (400 MHz, CDCl$_3$), ^{13}C NMR (101 MHz, CDCl$_3$), and GC-MS spectra of the product from Table 2, entry 4.

Analysis of reaction products referring to Table 2, entries 5 and 3: 1: 2,6-

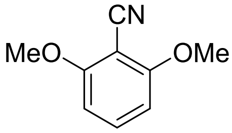

Dimethoxybenzonitrile: The product was isolated as white solid by column chromatography using 10-50 % ethyl acetate in hexane (EtOAc/Hex) as the eluent. The analytical data obtained matched the values reported in the literature.[11] ^{1}H NMR (400 MHz, $CDCl_3$) 7.41-7.45 (t, J = 8.5 Hz, 1H), 6.54-6.56 (d, J = 8.5 Hz, 2H), 3.91 (s, 3H). m/z calculated for $[M^+]$: 163.06; found: 163.1.

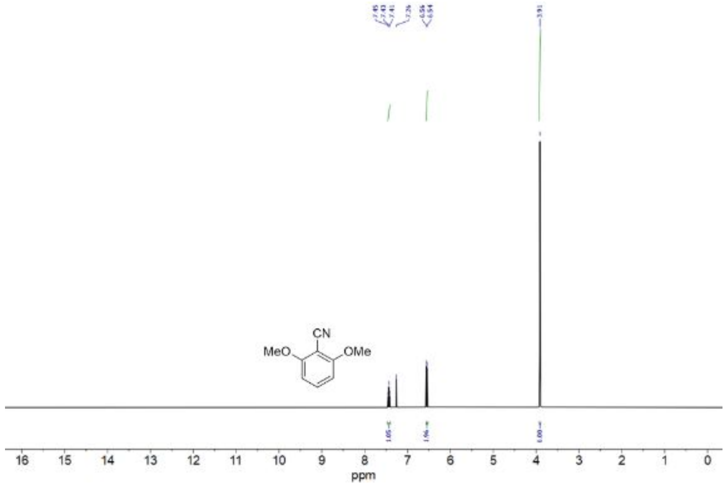

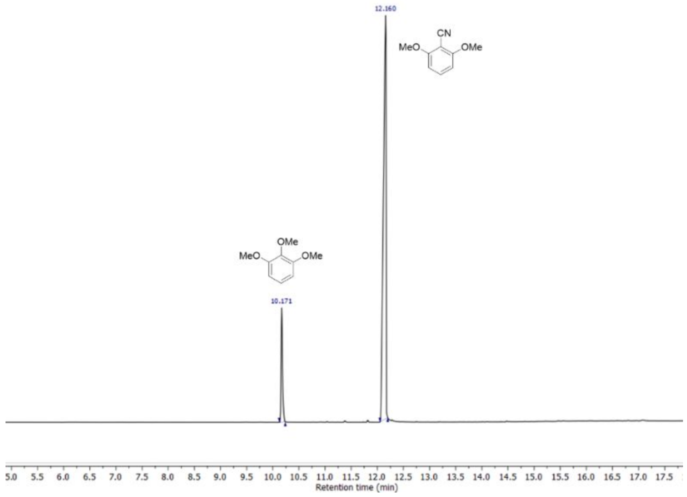

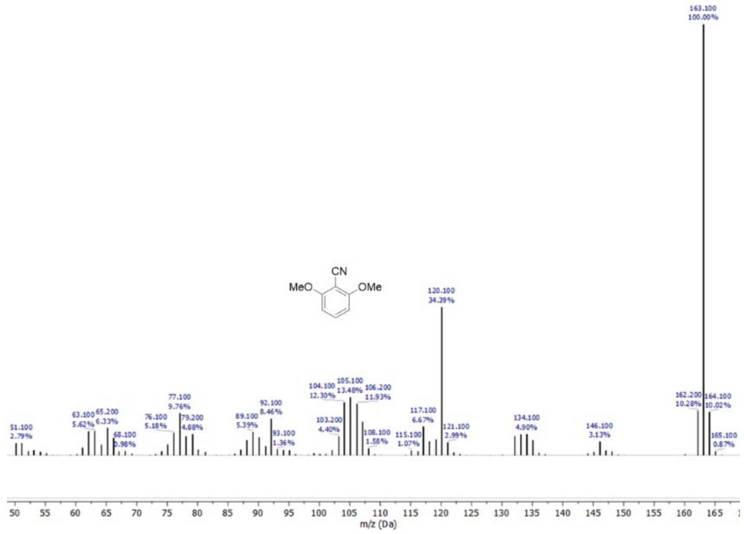

Figure S14. ^{1}H NMR (400 MHz, $CDCl_3$) and GC-MS spectra of the product from Table 2, entries 5 and 3.

Analysis of reaction products referring to Table 2, entry 6: 3-Chloro-4-methoxybenzonitrile:

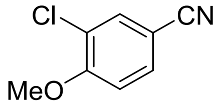

The product was isolated as a yellowish oil by column chromatography using 10% ethyl acetate in hexane (EtOAc/Hex) as the eluent. The analytical data obtained matched the values reported in the literature.[4,10] ^{1}H NMR (400 MHz, $CDCl_3$) δ 7.6 (d, J = 2.0 Hz, 1H), 7.5 (dd, J = 8.6, 2.0 Hz, 1H), 6.9 (d, J = 8.6 Hz, 1H), 3.9 (s, 3H). *m/z* calculated for [M$^+$]: 167.01; found: 167.1.

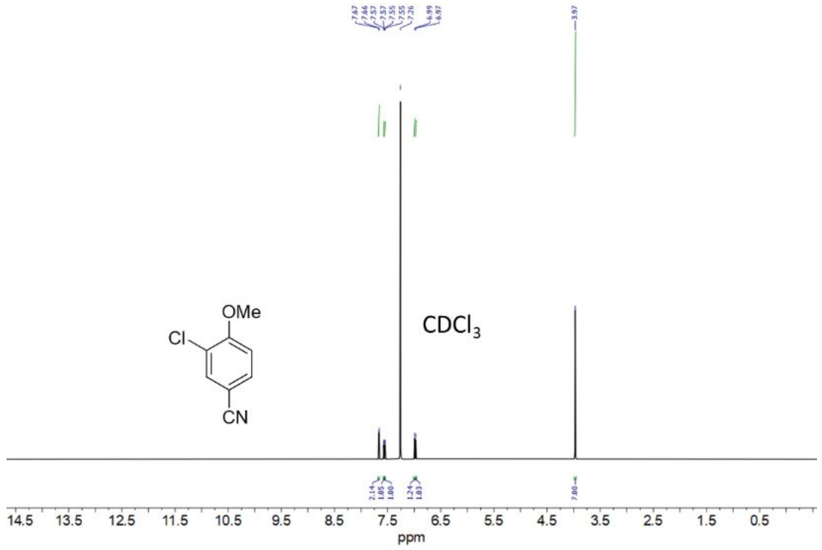

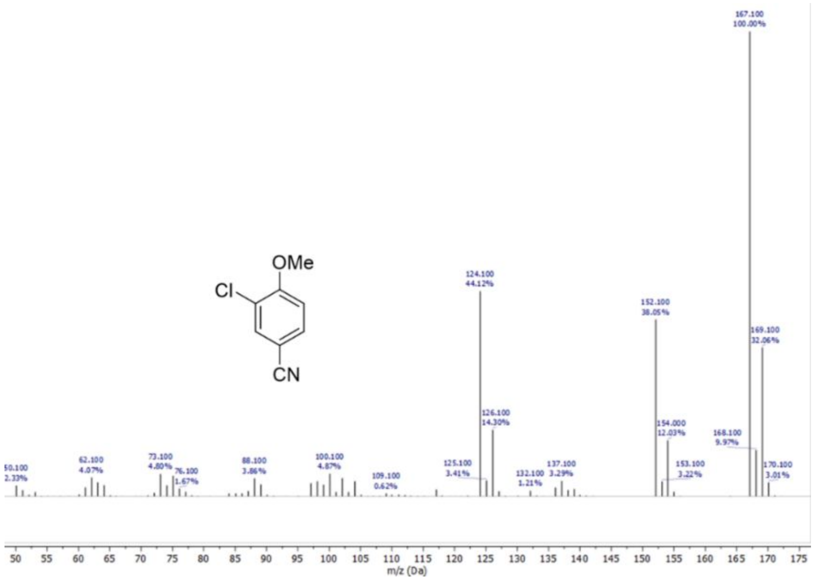

Figure S15. ^{1}H NMR (400 MHz, $CDCl_3$) and GC-MS spectra of the product from Table 2, entry 6.

Analysis of reaction products referring to Table 2, Entry 7: Mix of 2-methoxybenzonitrile compound with 3-fluoro-4-methoxybenzonitrile:

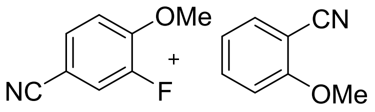

The product was isolated as mixture by column chromatography using 5-15 % EtOAc/Hex as the eluent. The analytical data obtained matched the values reported in the literature.[13,14] *m/z* calculated for [M^+]: 151.04 and 133.05; found: 151.1 and 133.1. ^{1}H NMR (400 MHz, $CDCl_3$) δ 7.44 (d, *J* = 8.5, 1H), 7.36 (d, *J* = 10.6, 2.0 Hz, 1H), 7.04-7.00 (m, 1H), 3.95 (s, 3H). ^{1}H NMR (400 MHz, $CDCl_3$) δ 7.58-7.51 (m, 2H), 7.02-6.98 (m, 2H), 3.94 (s, 3H).

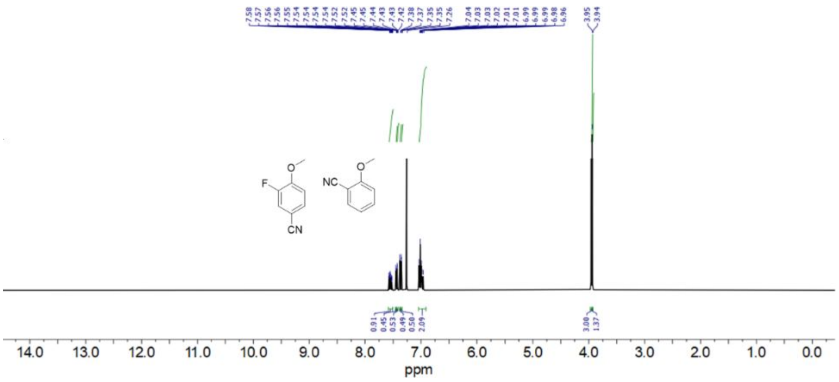

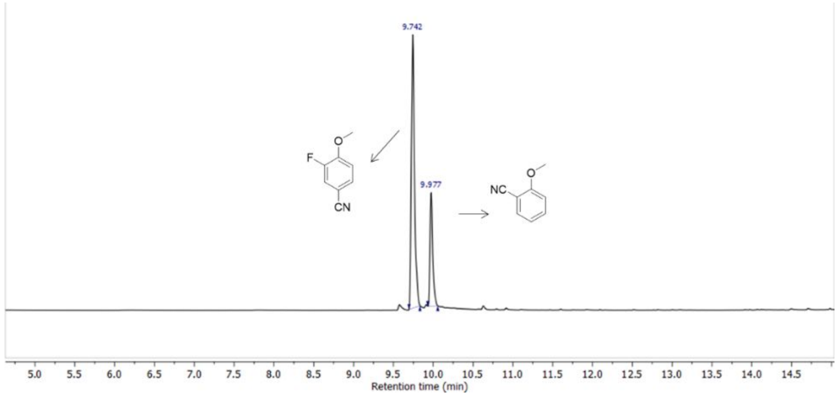

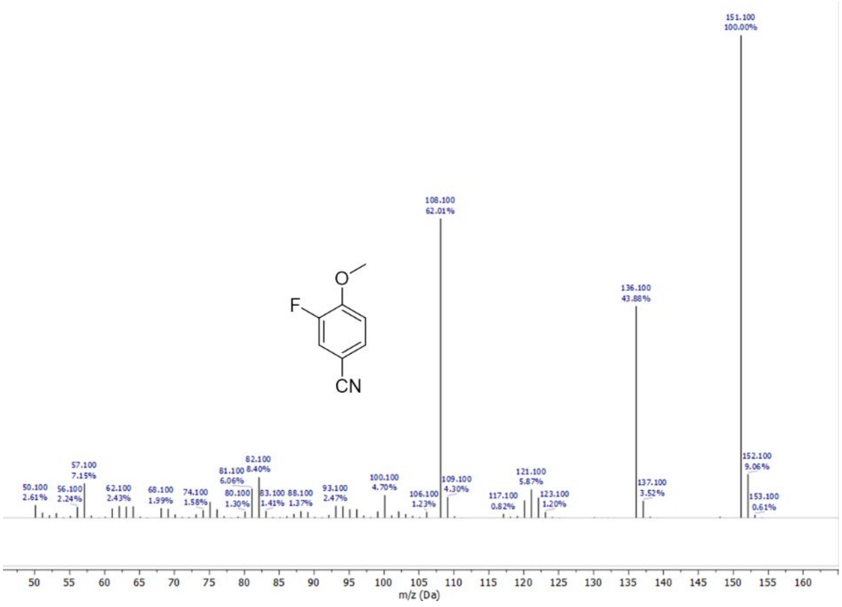

Figure S16. ^{1}H NMR (400 MHz, $CDCl_3$) and GC-MS spectra of the product from Table 2, entry 7.

Table S2. Scope of arene cyanation reaction by PMG_{100}-*b*-PPG_{38}/Mes-Acr-Ph^+ micelles in water.

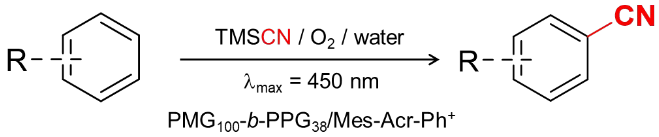

Entry [a]	Substrate	Detected Products	Time (h)	Conversion (%) [b]
1	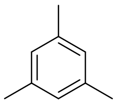	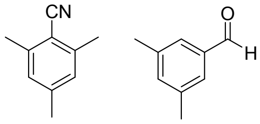	48	Trace [e]
2	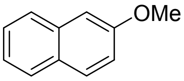	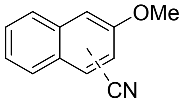	48	Trace [e]
3	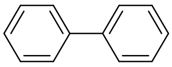	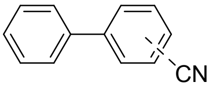	48	Trace [e]

[a] Reactions were conducted using 5 mL of a 0.2 mg/mL solution of PMG_{100}-*b*-PPG_{38}/Mes-Acr-Ph^+, arene substrate with concentration of 0.06 mM and molar ratio TMSCN/arene = 1.3. Irradiation with LED lamp (λ_{max} = 450 nm). O_2 purged for 15 min. [b] Conversion was obtained from the crude reaction mixture by ^{1}H NMR spectroscopy. [e] Detectable only by GC-MS.

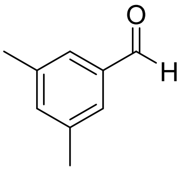

Product characterized corresponds to Table S2, entry 1: 3,5-dimethylbenzaldehyde Analytical data matched that reported in the literature.[15] *m/z* calculated for [M^+]: 145.1 and 134.1 found: 145.1 and [133.1 (100%) and 134.1 (86%)].

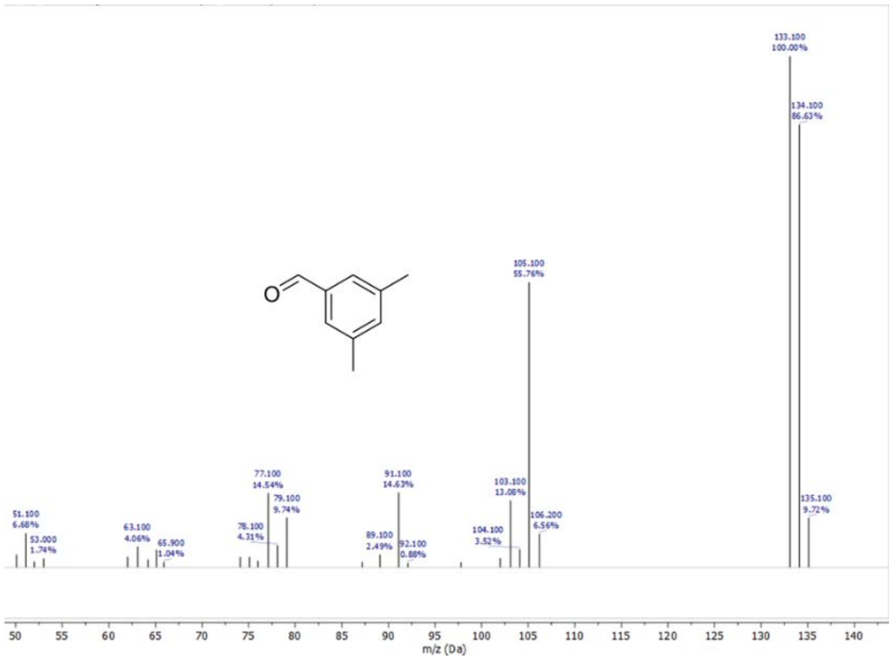

Figure S17. GC-MS spectrum of the product obtained from Table S2, entry 1.

Product characterized corresponds to Table S2, entry 2:

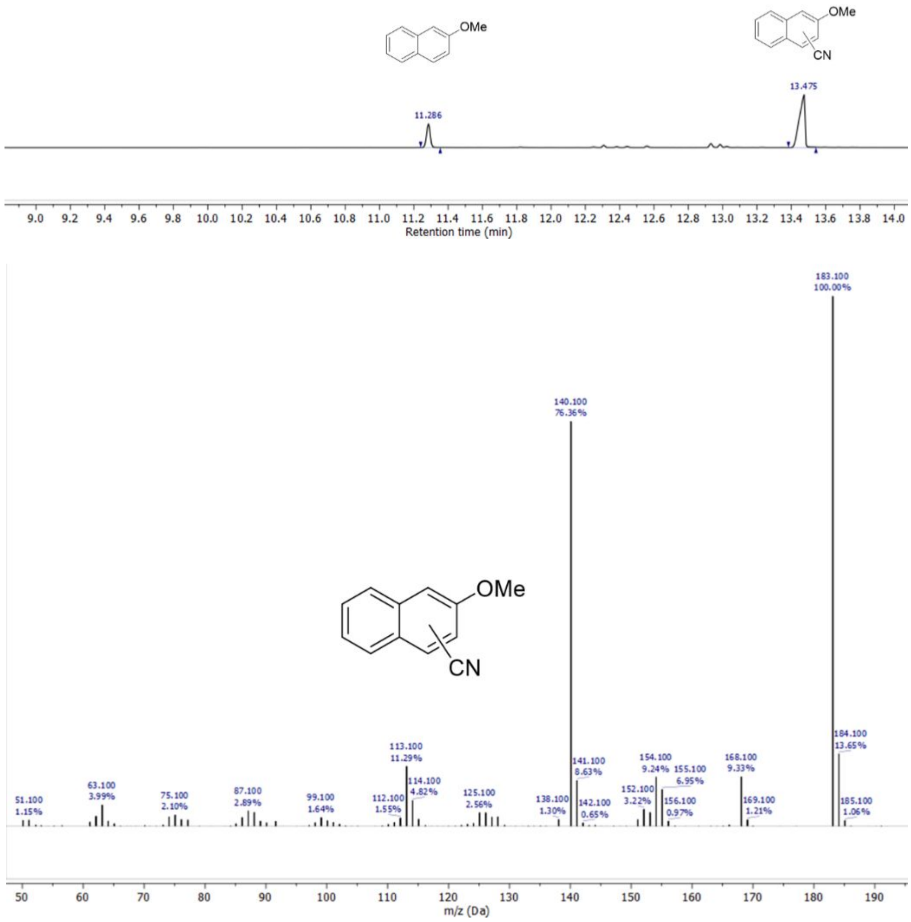

Figure S18. GC-MS spectrum of the product obtained from Table S2, entry 2.

Product characterized corresponds to Table S2, entry 3:

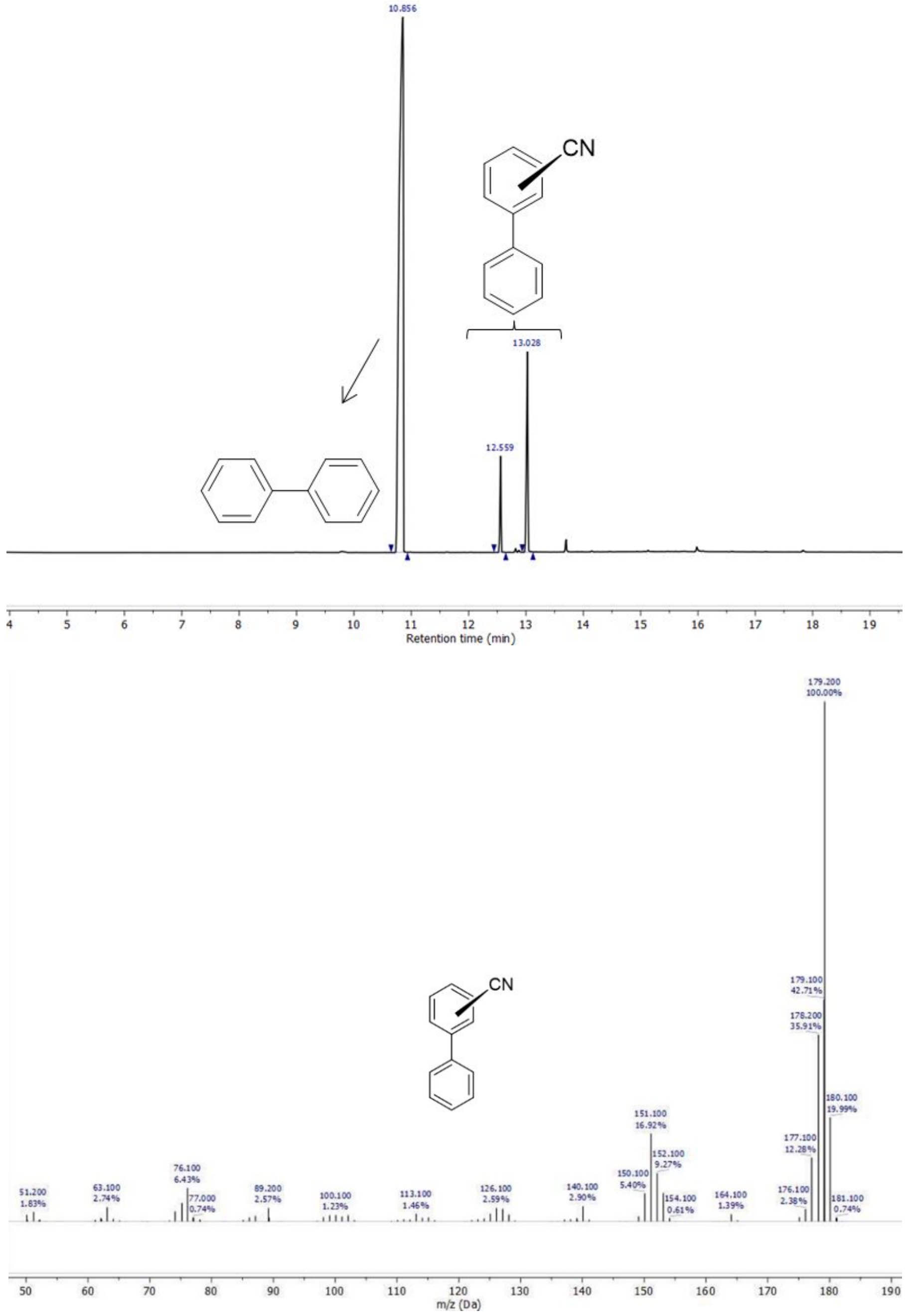

Figure S19. GC-MS spectrum of the product obtained from Table S2, entry 3.

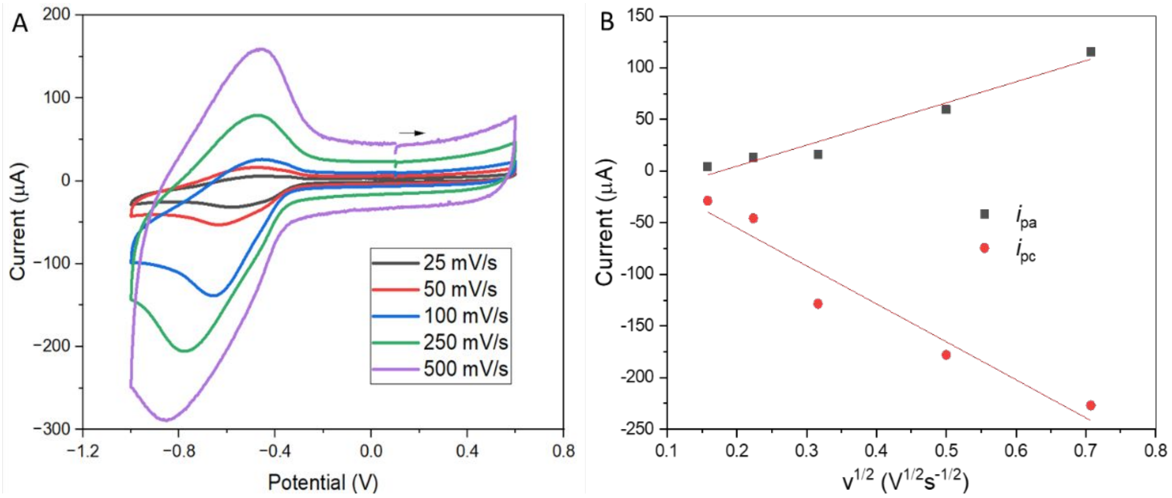

Figure S20. (A) Cyclic voltammograms of PMG_{100}-*b*-PPG_{38}/Mes-Acr-Ph^+ with various scan rates in water (potential range: −0.1 to +0.6 V (vs. Ag/AgCl)). (B) Dependence of anodic and cathodic peak currents (i_{pa}, i_{pc}) on the square root of the scan rate ($v^{1/2}$).

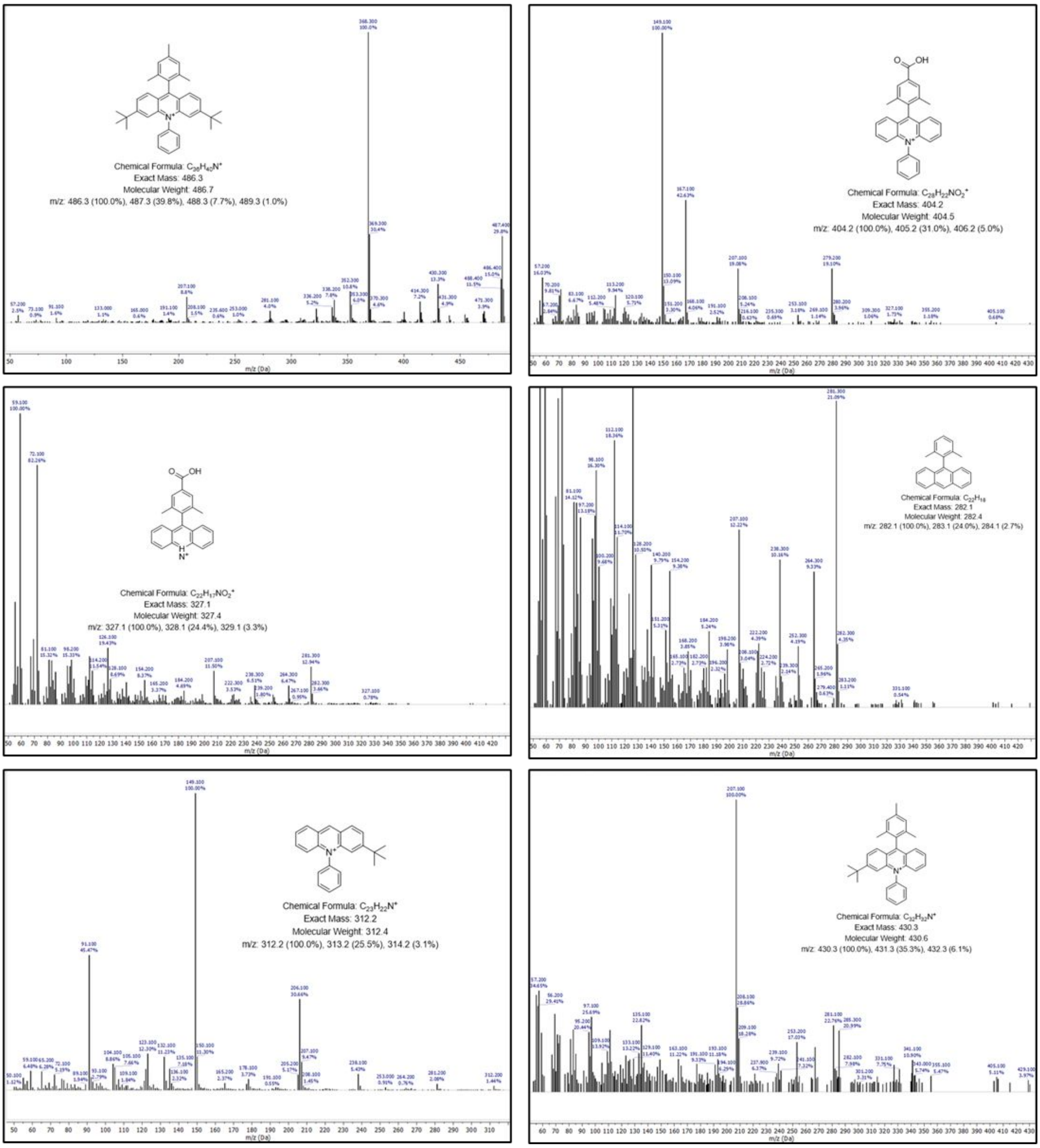

Figure S21. GC-MS analysis of the post-reaction mixtures from experiments shown in Tables 1 and 2, revealing species associated with Mes-Acr-Ph⁺ degradation.

References:

(1) Fetsch, C.; Grossmann, A.; Holz, L.; Nawroth, J. F.; Luxenhofer, R. Polypeptoids from N-Substituted Glycine n-Carboxyanhydrides: Hydrophilic, Hydrophobic, and Amphiphilic Polymers with Poisson Distribution. *Macromolecules* **2011**, *44* (17), 6746–6758.

(2) Robinson, J. W.; Secker, C.; Weidner, S.; Schlaad, H. Thermoresponsive Poly(N-C3 Glycine)s. Macromolecules **2013**, *46* (3), 580-587.

(3) Secker, C.; Völkel, A.; Tiersch, B.; Koetz, J.; Schlaad, H. Thermo-Induced Aggregation and Crystallization of Block Copolypeptoids in Water. *Macromolecules* **2016**, *49* (3), 979–985.

(4) McManus, J. B.; Nicewicz, D. A.; Singh, P. P.; Singh, J.; Srivastava, V. Direct C-H Cyanation of Arenes via Organic Photoredox Catalysis. *Journal of the American Chemical Society* **2017**, *139* (8), 2880–2883.

(5) Costabel, D.; Skabeev, A.; Nabiyan, A.; Luo, Y.; Max, J. B.; Rajagopal, A.; Kowalczyk, D.; Dietzek, B.; Wächtler, M.; Görls, H. 1, 7, 9, 10-Tetrasubstituted PMIs Accessible through Decarboxylative Bromination: Synthesis, Characterization, Photophysical Studies, and Hydrogen Evolution Catalysis. *Chemistry–A European Journal* **2021**, *27* (12), 4081–4088.

(6) Kowalczyk, D.; Li, P.; Abbas, A.; Eichhorn, J.; Buday, P.; Heiland, M.; Pannwitz, A.; Schacher, F. H.; Weigand, W.; Streb, C. Making Photocatalysis Comparable Using a Modular and Characterized Open-Source Photoreactor. *ChemPhotoChem* **2022**, *6* (7), e202200044.

(7) Sakamoto, M.; Sano, T.; Fujita, S.; Ando, M.; Yamaguchi, K.; Mino, T.; Fujita, T. Regioselective Photocycloaddition of Pyridine Derivatives to Electron-Rich Alkenes. *The Journal of Organic Chemistry* **2003**, *68* (4), 1447–1450.

(8) Zhang, G.; Ren, X.; Chen, J.; Hu, M.; Cheng, J. Copper-Mediated Cyanation of Aryl Halide with the Combined Cyanide Source. *Organic Letters* **2011**, *13* (19), 5004–5007.

(9) Yan, Y.; Sun, S.; Cheng, J. Palladium-Catalyzed Safe Cyanation of Aryl Iodides with Hexamethylenetetramine. *The Journal of Organic Chemistry* **2017**, *82* (23), 12888–12891.

(10) Shen, T.; Wang, T.; Qin, C.; Jiao, N. Silver-Catalyzed Nitrogenation of Alkynes: A Direct Approach to Nitriles through C≡C Bond Cleavage. *Angewandte Chemie International Edition* **2013**, *125* (26), 6677–6680.

(11) Liu, L.; Yeung, K.; Yu, J. Ligand-promoted Non-directed C− H Cyanation of Arenes. *Chemistry–A European Journal* **2019**, *25* (9), 2199–2202.

(12) Zhu, Y.; Zhao, M.; Lu, W.; Li, L.; Shen, Z. Acetonitrile as a Cyanating Reagent: Cu-Catalyzed Cyanation of Arenes. *Organic Letters* **2015**, *17* (11), 2602–2605.

(13) Yin, W.; Wang, C.; Huang, Y. Highly Practical Synthesis of Nitriles and Heterocycles from Alcohols under Mild Conditions by Aerobic Double Dehydrogenative Catalysis. *Organic Letters* **2013**, *15* (8), 1850–1853.

(14) Yu, H.; Richey, R. N.; Miller, W. D.; Xu, J.; May, S. A. Development of Pd/C-Catalyzed Cyanation of Aryl Halides. *The Journal of Organic Chemistry* **2011**, *76* (2), 665–668.

(15) Anbarasan, P.; Neumann, H.; Beller, M. A Convenient Synthesis of Benzonitriles via Electrophilic Cyanation with N-Cyanobenzimidazole. *Chemistry–A European Journal* **2010**, *16* (16), 4725–4728.
